# An optimized thymine base editing toolkit with various editing windows enables targeted T‐to‐G base conversions in rice

**DOI:** 10.1111/pbi.14611

**Published:** 2025-02-17

**Authors:** Xinbo Li, Yifu Tian, Rundong Shen, Yongzhen Pang, Kexuan Tang

**Affiliations:** ^1^ Yazhouwan National Laboratory Sanya Hainan China; ^2^ Hainan Seed Industry Laboratory Sanya Hainan China

**Keywords:** CRISPR, Cas‐embedding, TDG, base editing, rice

Base editors offer potential for site saturation mutagenesis, yet deaminase‐based editors are constrained to adenine and cytosine targets (Li *et al*., 2023a). Recently, glycosylase‐based base editors (gBEs), which fuse engineered glycosylases with SpCas9 nickase (SpCas9n, D10A) to excise specific guanine or thymine bases, achieve base conversions through DNA repair over abasic sites (He *et al*., 2024; Tong *et al*., 2023; Ye *et al*., 2024). While glycosylase‐based guanine base editors (gGBEs) show efficient guanine conversion in plants (Liu *et al*., 2024; Tian *et al*., 2024), thymine base editors (TBEs) remain unexplored (Figure S1).

Previous studies identified that the Y147A mutation in human uracil DNA glycosylase (hUNG) produces a thymine DNA glycosylase variant (hTDG). Highly active variants, TDG‐EK (He *et al*., 2024) and TDG3 (Ye *et al*., 2024), were engineered using protein‐language‐assisted design and directed evolution, respectively, to enhance thymine editing. Cas9‐embedding strategy further enhances base editing efficiency in mammalian cells (Figure 1a; He *et al*., 2024). To engineer efficient TBE tools for plants, we inserted three plant‐codon‐optimized TDG variants (hTDG, TDG‐EK and TDG3; Figure 1b) into SpCas9n at various positions (I1029‐G1030, F1046‐I1063 and P1249‐E1250) with a GGGGS linker.

**Figure 1:**
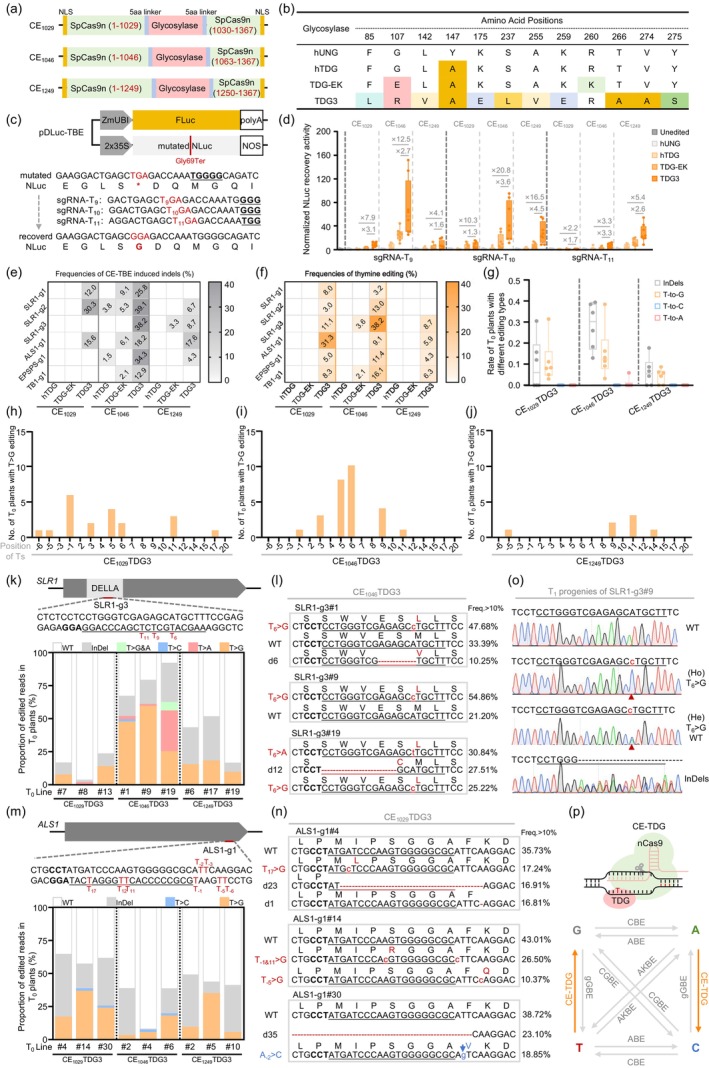
Engineering an efficient thymine base editing toolkit in rice. (a) Schematic of Cas‐embedded base editors. (b) Glycosylase variants used in this study. (c) Dual‐luciferase reporter system for assessing thymine editing in rice protoplasts. (d) NLuc restoration activities of different glycosylase base editors. The average relative luminescence units restored by CE_1029_hTDG are normalized to 1 (*n* = 8). (e, f) Indel (e) and thymine base editing (f) efficiencies of CE‐TBEs across six targets. Heat maps show ratios of edited T_0_ plants. (g) Editing types and efficiencies of CE‐TDG3 at six endogenous targets. (h–j) Summary of base‐edited rice T_0_ plantlets across the protospacers from six endogenous targets edited by CE_1029_TDG3 (h), CE_1046_TDG3 (i), and CE_1249_TDG3 (j). Plants with read proportions >10% in Hi‐TOM sequencing were counted. (k–n) Mutations and proportions in T_0_ plants at SLR1‐g3 (k) and ALS1‐g1 (m), analysed using Hi‐TOM. Sequencing reads and their proportions in representative T_0_ plants targeted at SLR1‐g3 by CE_1046_TDG3 (l) and at ALS1‐g1 by CE_1029_TDG3 (n). (o) Genotyping results of T_1_ plants. (p) Diagram of base conversions using available editors in rice.

Initially, we assessed the thymine editing efficiency of three TDG variants (hTDG, TDG‐EK and TDG3) in rice protoplasts using a dual‐luciferase reporter system, with hUNG as a control. The system utilized *ZmUBI*‐driven firefly luciferase (FLuc) as a reference and *2x35S*‐driven NanoLuc (NLuc) as the reporter. A nonsense mutation was introduced at Gly69 (GGA > TGA), which was converted to a read‐through codon by successful thymine editing, restoring NLuc translation (Figure S2). Three sgRNAs were designed to edit specific thymine positions (T9, T10 and T11; Figure 1c). Transient assays revealed that TDG3 outperformed hTDG by 2.2‐ to 20.8‐fold and TDG‐EK by 1.3‐ to 4.5‐fold, with CE_1046_TDG3 showing the highest activity at T9 (Figure 1d, Figure S3).

To evaluate editing efficiencies of Cas9‐embbeded TBEs (CE‐TBEs) in transgenic rice plants, we tested six endogenous targets (SLR1‐g1, SLR1‐g2, SLR1‐g3, ALS1‐g1, EPSPS‐g1, TB1‐g1; Table S1). A total of 1681 T_0_ plants edited by nine CE‐TBEs were regenerated and analysed via Hi‐TOM sequencing (Table S2). Plants with chimerism >10% were considered valid edits (Figure S4) and confirmed by subcloning sequencing (Figure S5). Genotyping revealed that the hTDG variant induced only 3.8% and 1.5% indels at SLR1‐g2 and ALS1‐g1, respectively (Figure 1e).

For the TDG‐EK variant, the CE_1029_TDG‐EK and CE_1249_TDG‐EK constructs showed no detectable thymine editing activity, while CE_1046_TDG‐EK achieved T‐to‐G transversion at T11 of SLR1‐g3 and T3 of TB1‐g1 with 3.6% (1/28) and 2.1% (1/48), respectively (Figure 1f). Consistent with protoplast results, the TDG3 variant significantly enhanced glycosylase activity in rice, increasing thymine editing and indel efficiencies (Figure 1e,f). CE_1046_TDG3 outperformed CE_1029_TDG3 and CE_1249_TDG3, with average efficiencies of 29.1% for indels and 16.4% for thymine editing (Figure 1g). The highest thymine editing efficiency was 38.2% (13/34) at SLR1‐g3 (Figure 1f).

The CE‐TDG3 constructs predominantly yielded T‐to‐G editing in rice T_0_ plants, except for CE_1046_TDG3, which induced 5.9% (2/34) T‐to‐A editing at the SLR1‐g3 target. No T‐to‐C editing was detected. Notably, different embedding variants exhibited distinct activity windows. CE_1029_TDG3 showed a wide editable range (T‐6 to T17; with the PAM located at 21–23; Figure 1h), while CE_1046_TDG3 displayed a narrower editing window (T‐1 to T11; Figure 1i). The CE_1249_TDG3 led to a backward‐shifted editing window (mainly T9–T14; Figure 1j).

At the SLR3‐g3 target, plants with a high proportion of amino acid substitutions or in‐frame deletions at the TVHYNP motif exhibit a semi‐dwarf phenotype (Figure 1k,l, Figure S6). Notably, T‐to‐G conversions were observed within protospacers at all tested targets except ALS1‐g1, where CE_1029_TDG3, CE_1046_TDG3 and CE_1249_TDG3 induced high proportions of thymine mutations outside the protospacer or on the targeted strand (Figure 1m,n, Table S2). Although most edits were chimeric, likely due to sustained CE‐TDG3 activity, T‐DNA‐free T‐to‐G mutants were obtained in the T_1_ generation (Figure 1o, Table S4).

In this study, we compared a series of Cas9‐embedded glycosylase constructs and developed three efficient TBEs: CE_1029_TDG3, CE_1046_TDG3 and CE_1249_TDG3. Genotyping of 1681 T_0_ plants demonstrated that CE‐TDG3 constructs enabled targeted T‐to‐G editing within distinct activity windows, achieving up to 38.2% efficiency in rice. Unlike editing in mammalian cells (predominantly T‐to‐C/G; He *et al*., 2024) and *Escherichia coli* (mainly T‐to‐A; Ye *et al*., 2024), our rice‐optimized TBE (CE‐TDG3) primarily induced T‐to‐G editing, with no significant off‐target activity (Table S5). Improving TDG activity would further enhance editing efficiency and purity (Tong *et al*., 2024). Combining CE‐TDG3 with ABE and AKBE (Li *et al*., 2023b; Tan *et al*., 2022; Wu *et al*., 2023) allows the conversion of T:A base‐pair to any desired base‐pair (Figure 1p), broadening possibilities for generating elite germplasm.

## Author contributions

Y.T. and X.L. designed the research; X.L., Y.T. and R.S. performed the experiments; Y.T. and X.L analysed the data; Y.T., Y.P. and K.T. wrote and revised the manuscript.

## Conflict of interest

The authors declare no competing interests.

## Supporting information


**Figure S1** Composition of current base editors.
**Figure S2** Identification of editing results on *NLuc*.
**Figure S3** Comparison of editing activity of TBEs with TDG3 fusion at different positions.
**Figure S4**. Representative amplicon sequencing results of selected T_0_ plants.
**Figure S5** Representative subcloning sequencing results of selected T_0_ plants.
**Figure S6** Representative phenotype of selected plants.
**Sequence S1** DNA sequences of related vectors and genes.
**Sequence S2** DNA sequence of the NLuc cassette of pDLuc‐TBE.
**Table S1** Summary of the targeted sites.
**Table S2** Summary of CE‐TBEs induced editing in rice T_0_ plants.
**Table S3** Summary of HiTOM sequencing results of T_0_ plants.
**Table S4** Summary of the off‐target sites tested in this study.
**Table S5** Heritability analysis on T1 progenies.
**Table S6** Primers and oligos used in this study.

## Data Availability

The plasmids (CE1029hTDG, CE1029TDG‐EK, CE1029TDG3, CE1046hTDG, CE1046TDG‐EK, CE1046TDG3, CE1249hTDG, CE1249TDG‐EK, CE1249TDG3, NTDG3 and CTDG3) constructed in this study are available upon request to corresponding author (tianyifu@caas.cn). The NGS data was deposited in the National Genomics Data Center (NGDC; https://ngdc.cncb.ac.cn/) under the accession number PRJCA030169 and PRJCA034519.

## References

[pbi14611-bib-0001] He, Y. , Zhou, X. , Chang, C. , Chen, G. , Liu, W. , Li, G. , Fan, X. *et al*. (2024) Protein language models‐assisted optimization of a uracil‐N‐glycosylase variant enables programmable T‐to‐G and T‐to‐C base editing. Mol. Cell, 84(7), 1257–1270.e6. 38377993 10.1016/j.molcel.2024.01.021

[pbi14611-bib-0002] Li, J. , Zhang, C. , He, Y. , Li, S. , Yan, L. , Li, Y. , Zhu, Z. *et al*. (2023a) Plant base editing and prime editing: the current status and future perspectives. J. Integr. Plant Biol. 65(2), 444–467. 36479615 10.1111/jipb.13425

[pbi14611-bib-0003] Li, Y. , Li, S. , Li, C. , Li, S. , Yan, L. , Li, Y. , Zhu, Z. *et al*. (2023b) Engineering a plant A‐to‐K base editor with improved performance by fusion with a transactivation module. Plant Commun. 4(6), 100667. 37528582 10.1016/j.xplc.2023.100667 PMC10721455

[pbi14611-bib-0004] Liu, L. , Zhang, Z. , Wang, C. , Yan, F. , Sun, W. , Zhou, X. , Miao, W. *et al*. (2024) Developing guanine base editors for G‐to‐T editing in rice. J. Integr. Plant Biol. 66(8), 1557–1560. 38934772 10.1111/jipb.13729

[pbi14611-bib-0005] Tan, J. , Zeng, D. , Zhao, Y. , Wang, Y. , Liu, T. , Li, S. , Xue, Y. *et al*. (2022) PhieABEs: a PAM‐less/free high‐efficiency adenine base editor toolbox with wide target scope in plants. Plant Biotechnol. J. 20(5), 934–943. 34984801 10.1111/pbi.13774 PMC9055815

[pbi14611-bib-0006] Tian, Y. , Li, X. , Xie, J. , Zheng, Z. , Shen, R. , Cao, X. , Wang, M. *et al*. (2024) Targeted G‐to‐T base editing for generation of novel herbicide‐resistance gene alleles in rice. J. Integr. Plant Biol. 66(6), 1048–1051. 38578176 10.1111/jipb.13657

[pbi14611-bib-0007] Tong, H. , Liu, N. , Wei, Y. , Zhou, Y. , Li, Y. , Wu, D. , Jin, M. *et al*. (2023) Programmable deaminase‐free base editors for G‐to‐Y conversion by engineered glycosylase. Natl. Sci. Rev. 10(8), nwad143. 37404457 10.1093/nsr/nwad143 PMC10317176

[pbi14611-bib-0008] Tong, H. , Wang, H. , Wang, X. , Liu, N. , Li, G. , Wu, D. , Li, Y. *et al*. (2024) Development of deaminase‐free T‐to‐S base editor and C‐to‐G base editor by engineered human uracil DNA glycosylase. Nat. Commun. 15(1), 4897. 38851742 10.1038/s41467-024-49343-5 PMC11162499

[pbi14611-bib-0009] Wu, X. , Ren, B. , Liu, L. , Qiu, S. , Li, X. , Li, P. , Yan, F. *et al*. (2023) Adenine base editor incorporating the N‐methylpurine DNA glycosylase MPGv3 enables efficient A‐to‐K base editing in rice. Plant Commun. 4(6), 100668. 37528583 10.1016/j.xplc.2023.100668 PMC10721470

[pbi14611-bib-0010] Ye, L. , Zhao, D. , Li, J. , Wang, Y. , Li, B. , Yang, Y. , Hou, X. *et al*. (2024) Glycosylase‐based base editors for efficient T‐to‐G and C‐to‐G editing in mammalian cells. Nat. Biotechnol. 42, 1538–1547. 38168994 10.1038/s41587-023-02050-w

