# Supplementary material for: An optimized thymine base editing toolkit with various editing windows enables targeted T‐to‐G base conversions in rice
Source: Plant Biotechnol J. 2025 Feb 17;23(5):1637–9. doi: 10.1111/pbi.14611 (PMC12018829; doi:10.1111/pbi.14611)
Supplement: Supplementary file 1 — Figure S1 Composition of current base editors. Figure S2 Identification of editing results on NLuc. Figure S3 Comparison of editing activity of TBEs with TDG3 fusion at different positions. Figure S4. Representative amplicon sequencing results of selected T0 plants. Figure S5 Representative subcloning sequencing results of selected T0 plants. Figure S6 Representative phenotype of selected plants. Sequence S1 DNA sequences of related vectors and genes. Sequence S2 DNA sequence of the NLuc cassette of pDLuc‐TBE. Table S1 Summary of the targeted sites. Table S2 Summary of CE‐TBEs induced editing in rice T0 plants. Table S3 Summary of HiTOM sequencing results of T0 plants. Table S4 Summary of the off‐target sites tested in this study. Table S5 Heritability analysis on T1 progenies. Table S6 Primers and oligos used in this study. [file PBI-23-1637-s001.docx]

### Supplementary figures and tables for

**An optimized thymine base editing toolkit with various editing windows enable targeted T-to-G base conversions in rice**

Xinbo Li^1,2,3^, Yifu Tian^1,2,3^*, Rundong Shen^1^, Yongzhen Pang^1^, Kexuan Tang^1^

^1^Yazhouwan National Laboratory, Sanya, Hainan 572024, China.

^2^Hainan Seed Industry Laboratory, Sanya, Hainan 572024, China.

^3^These authors contributed equally.

*Correspondence: tianyifu@caas.cn.

**Running title:** Targeted T-to-G editing in rice.

**Keywords:** CRISPR, Cas-embedding, TDG, base editing, rice

**Methods and Materials**

**Plasmid construction.**

To construct Cas-embedded thymine base editor (CE-TBE) vectors, the hUNG and TDG3 variants were commercially synthesized (Genewiz, Suzhou, China), while hTDG and TDG-EK were generated via site-directed mutagenesis from hUNG. These glycosylase variants were inserted into the rice SpCas9 nickase (D10A) using overlapping PCR. CE-glycosylases were then cloned into the rice rABE8e vector (Wei et al., 2021) using the ClonExpressII One Step Cloning Kit (Vazyme, Nanjing, China; Supplementary Sequence 1). A VirD2-derived nuclear localization signal (Hua et al., 2018) was fused to the C-terminus of CE-TBEs to enhance nuclear import efficiency. The NTDG3 and CTDG3 were constructed by fusing the TDG3 variant to the N- and C-terminus of SpCas9n, respectively, using the ClonExpressII One Step Cloning Kit.

The pDLuc-TBE plasmid (Supplementary Sequence 2) was modified from the previously constructed pDual-LucM (Tian et al., 2022). A Gly69* (GGA>TGA) mutation was introduced into the NLuc gene using the ClonExpressII One Step Cloning Kit (Vazyme, Nanjing, China). To install the sgRNA expression cassettes, 23-bp target sequences, including the PAM (Supplementary Table 1), were selected and analyzed via CRISPR-P (Liu et al., 2017). The 20-bp spacers were inserted into CE-TBEs using Golden Gate cloning. Primers and oligos used in this study are listed in Supplementary Table 6 and were synthesized by Sangon Biotech.

**Protoplast transfection and dual-luciferase reporter assay**

Japonica rice variety Nipponbare was used for protoplast assay in this study. The rice seedlings were grown in the dark at 28°C for 10 days. Protoplast isolation and transformation followed previously described methods (Jin et al., 2023). After transfection, the protoplasts were incubated at 23°C in the dark for 24 hours. Luciferase activity was measured using the Dual-Glo™ Luciferase Assay System (Promega), following the manufacturer's protocol.

***Agrobacterium*-mediated transformation of rice callus cells**

*Agrobacterium tumefaciens* strain EHA105 was transformed with the binary vectors using the freezing/heat shock method. *Agrobacterium*-mediated transformation of callus cells of *Nipponbare* was conducted as described (Lu et al., 2017). Hygromycin B (50 mg/L, Shanghai YEASEN Biotechnology Co., Ltd.) was applied to select hygromycin-resistant calli. Resistant calli were subsequently regenerated into plantlets following standard rice transformation protocols (Nishimura et al., 2006).

**Genotyping**

To genotype the T_0_ transgenic lines, genomic DNA was extracted from leaves by using CTAB method. To accurately identify the types and proportions of editing outcomes, leaf tissues were harvested from three different tillers of each plant (~2 mg from each tiller) and pooled for DNA extraction. To assess the mutagenesis frequency, the targeted sequences of sgRNAs were amplified for HiTOM sequencing (Liu et al., 2019) and confirmed by Sanger sequencing. The editing efficiencies were determined with a 10% threshold in HiTOM assay (Supplementary Table 3). To assess the heritability of the editing results, T_1_ seedlings grown in the greenhouse for 2 weeks were sampled, and genomic DNA was extracted using the CTAB method. Genotyping was performed using Hi-TOM and/or Sanger sequencing.

**Off-target analysis**

To investigate off-target effects for all targets, we selected potential off-target sites based on predictions from CRISPR-GE (http://skl.scau.edu.cn/offtarget/, Xie et al., 2017). Hi-TOM sequencing were employed to assess off-target effects. All primer sets used in this study are listed in Supplemental table 6.

**Statistical analysis**

The relevant statistical test, sample size and replicate type for each figure and table are found in the figure or table and/or the corresponding figure legends.

**Data availability**

The plasmids (CE_1029_hTDG, CE_1029_TDG-EK, CE_1029_TDG3, CE_1046_hTDG, CE_1046_TDG-EK, CE_1046_TDG3, CE_1249_hTDG, CE_1249_TDG-EK, CE_1249_TDG3, NTDG3 and CTDG3) constructed in this study are available upon request to corresponding author (tianyifu@caas.cn). The NGS data was deposited in the National Genomics Data Center (NGDC; https://ngdc.cncb.ac.cn/) under the accession number PRJCA030169 and PRJCA034519.

**Supplementary references**

**Banno, S., Nishida, K., Arazoe, T., et al.** (2018). Deaminase-mediated multiplex genome editing in Escherichia coli. Nat. Microbiol., 3(4), 423–429.

**Gaudelli, N. M., Komor, A. C., Rees, H. A., et al.** (2017). Programmable base editing of A•T to G•C in genomic DNA without DNA cleavage. Nature, 551(7681), 464–471.

**He, Y., Zhou, X., Chang, C., et a.** (2024). Protein language models-assisted optimization of a uracil-N-glycosylase variant enables programmable T-to-G and T-to-C base editing. Mol. cell, 84(7), 1257–1270.e6.

**Hua, K., Tao, X., Yuan, F., et al.** (2018). Precise A·T to G·C Base Editing in the Rice Genome. Mol. plant, 11(4), 627–630.

**Jin, S., Lin, Q., Gao, Q., et al.** (2023). Optimized prime editing in monocot plants using PlantPegDesigner and engineered plant prime editors (ePPEs). Nat. Protoc. 18(3):831-853.

**Komor, A. C., Kim, Y. B., Packer, M. S., et al.** (2016). Programmable editing of a target base in genomic DNA without double-stranded DNA cleavage. Nature, 533(7603), 420–424.

**Liu, H., Ding, Y., Zhou, Y., et al.** (2017). CRISPR-P 2.0: An Improved CRISPR-Cas9 Tool for Genome Editing in Plants. Mol. Plant. 10(3):530-532.

**Liu, Q., Wang, C., Jiao, X., et al.** (2019). Hi-TOM: a platform for high-throughput tracking of mutations induced by CRISPR/Cas systems. Sci. China Life Sci. 62(1):1-7.

**Lu, Y., Tian, Y., Shen, R., et al.** (2020). Targeted, efficient sequence insertion and replacement in rice. Nat. Biotechnol. 38(12):1402-1407.

**Lu, Y., Ye, X., Guo, R., et al.** (2017). Genome-wide targeted mutagenesis in rice using the CRISPR/Cas9 system. Mol. Plant. 10(9):1242-1245.

**Nishimura, A., Aichi, I., Matsuoka, M.** (2006). A protocol for Agrobacterium-mediated transformation in rice. Nat. Protoc., 1(6), 2796–2802.

**Sretenovic, S., Liu, S., Li, G., et al.** (2021). Exploring C-To-G Base Editing in Rice, Tomato, and Poplar. Front. Genome Ed., 3, 756766.

**Tian, Y., Shen, R., Li, Z., et al.** (2022). Efficient C-to-G editing in rice using an optimized base editor. Plant Biotechnol. J. 20(7):1238-1240.

**Tian, Y., Li, X., Xie, J., et al.** (2024). Targeted G-to-T base editing for generation of novel herbicide-resistance gene alleles in rice. Journal of integrative plant biology, 66(6), 1048–1051.

**Tong, H., Wang, X., Liu, Y., et al.** (2023a). Programmable A-to-Y base editing by fusing an adenine base editor with an N-methylpurine DNA glycosylase. Nat. Biotechnol., 41(8), 1080–1084.

**Tong, H., Liu, N., Wei, Y., et a.** (2023b). Programmable deaminase-free base editors for G-to-Y conversion by engineered glycosylase. Natl. Sci. Rev., 10(8), nwad143.

**Wei, C., Wang, C., Jia, M., et al.** (2021) Efficient generation of homozygous substitutions in rice in one generation utilizing an rABE8e base editor. J. Integr. Plant Biol. 63(9):1595-1599.

**Wu, X., Ren, B., Liu, L., et al.** (2023). Adenine base editor incorporating the N-methylpurine DNA glycosylase MPGv3 enables efficient A-to-K base editing in rice. Plant Commun., 4(6), 100668.

**Xie, X., Ma, X., Zhu, Q., et al.** (2017). CRISPR-GE: A Convenient Software Toolkit for CRISPR-Based Genome Editing. Mol Plant. 2017 Sep 12;10(9):1246-1249.

**Ye, L., Zhao, D., Li, J., et al.** (2024). Glycosylase-based base editors for efficient T-to-G and C-to-G editing in mammalian cells. Nat. Biotechnol., 10.1038/s41587-023-02050-w. Advance online publication.

**Zong, Y., Wang, Y., Li, C., Zhang, R., et al.** (2017). Precise base editing in rice, wheat and maize with a Cas9-cytidine deaminase fusion. Nat. Biotechnol., 35(5), 438–440.

**Zhao, D., Li, J., Li, S., et al.** (2021). Glycosylase base editors enable C-to-A and C-to-G base changes. Nat. Biotechnol., 39(1), 35–40.


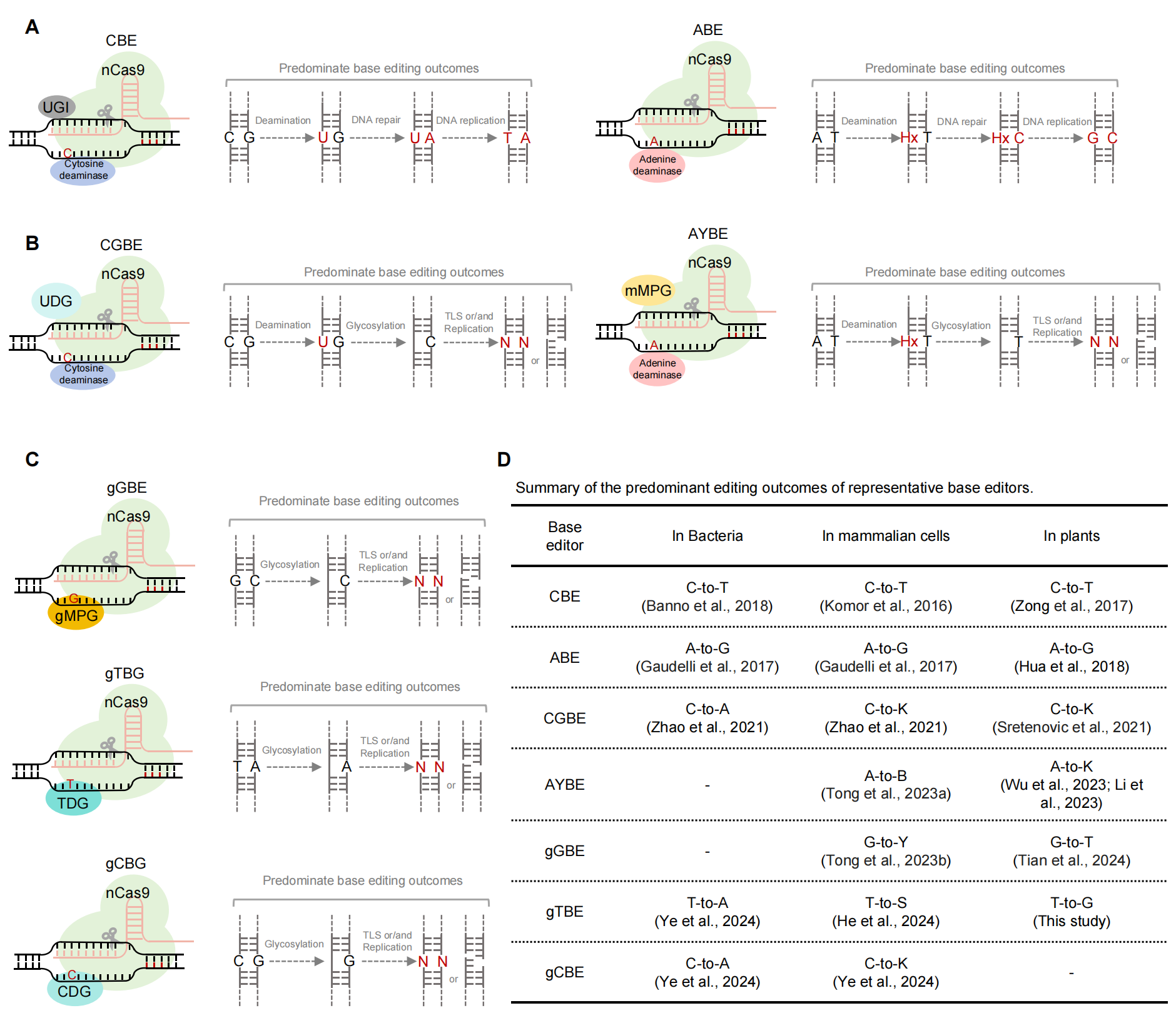


**Supplementary figure 1. Composition of current base editors.** **(A)** Cytosine base editors (CBEs) and adenine base editors (ABEs) are two categories of deaminase-based base editors, initiating base conversion via deamination of cytosine and adenine, respectively. The deaminated products uracil (U) and hypoxanthine (Hx) are recognized as thymine (T) and guanine (G) during DNA replication, thus inducing C-to-T and A-to-G base editing. **(B)** CGBEs (C-to-G base editors) and AYBEs (A-to-Y base editors) are constructed by incorporating uracil DNA glycosylase (UDG) and mutated N-methylpurine-DNA glycosylase (mMPG) in CBE and ABE, respectively, excising the uracil and hypoxanthine to produce abasic sites. The Subsequent DNA repair and replication over the abasic sites enabled both targeted base transition and transversion, expanding the repertoire of base editing products. **(C)** Glycosylase-based base editors are newly developed base editors by fusing specific glycosylase with Cas9 nickase (nCas9), thus directly excising targeted thymine (T), guanine (G), and cytosine (C) to promote base conversion via DNA repair and DNA replication. **(D)** Summary of the predominant editing outcomes of representative base editors in different organisms.


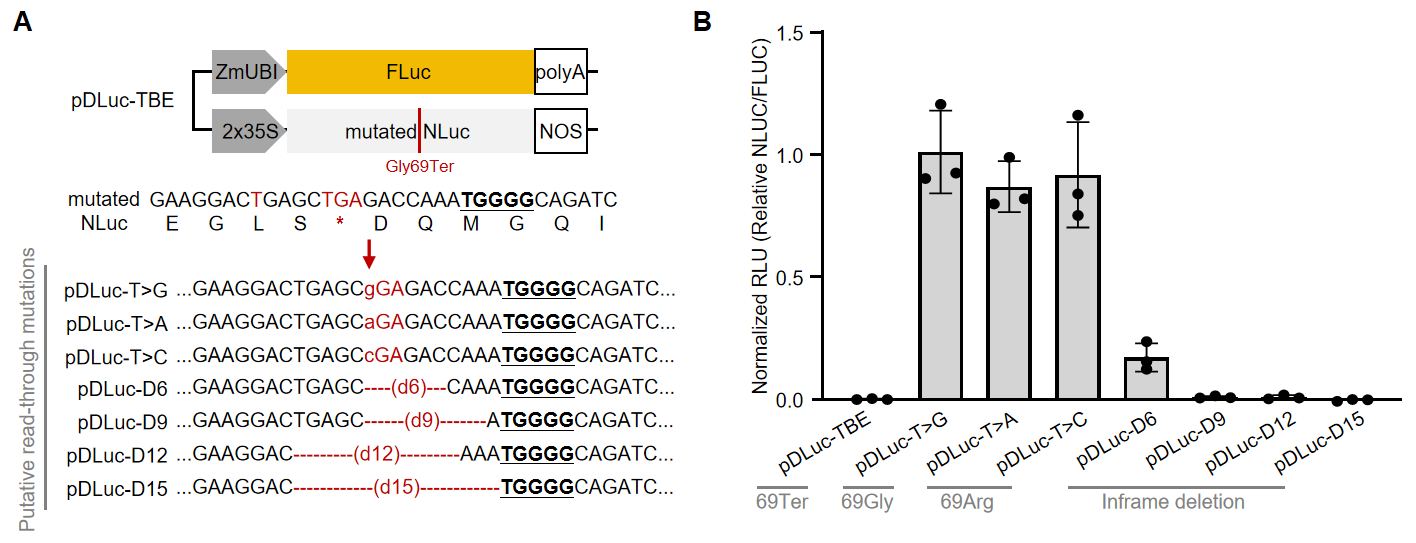


**Supplementary Figure 2. Identification of editing results on *NLuc*. (A)** Putative in-frame editing outcomes on mutated *NLuc* generated by plant TBEs. **(B)** Luciferase activity assay of read-through mutant products edited by TBEs (n=3). Mutations that replace the T base with other bases fully restored Nano luciferase activity, whereas in-frame InDels significantly reduced or even abolished Nano luciferase activity.


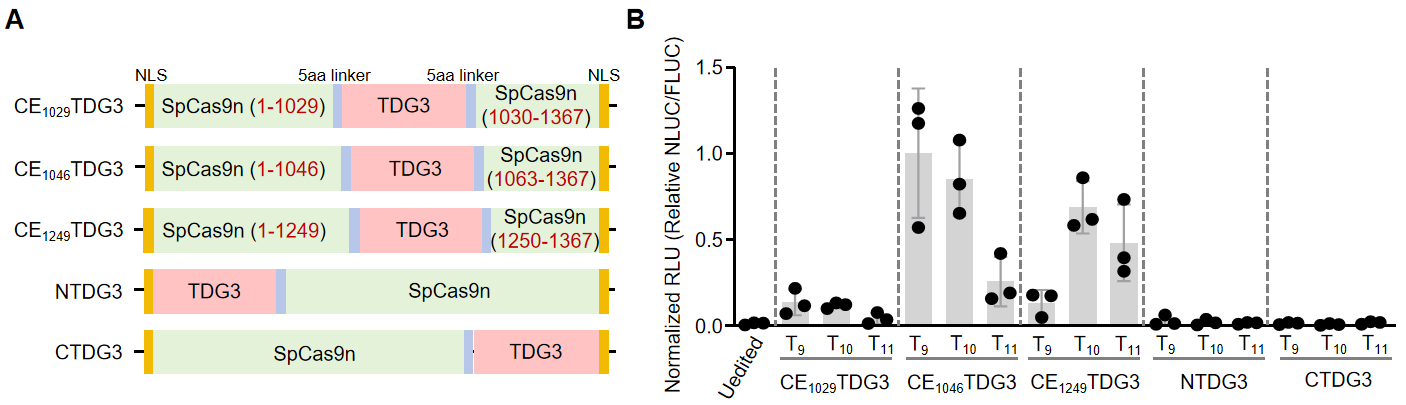


**Supplementary Figure 3. Comparison of editing activity of TBEs with TDG3 fusion at different positions. (A)** The schematic illustrates the configuration of TDG fused to either the N-terminus, C-terminus, or the middle of the nCas9 protein. **(B)** Comparison of relative editing activity of different TBE systems with varying TDG3 fusion positions, measured using the dual-luciferase reporter system (n=3). The average RLU of CE_1046_TDG3 was standardized to 1.


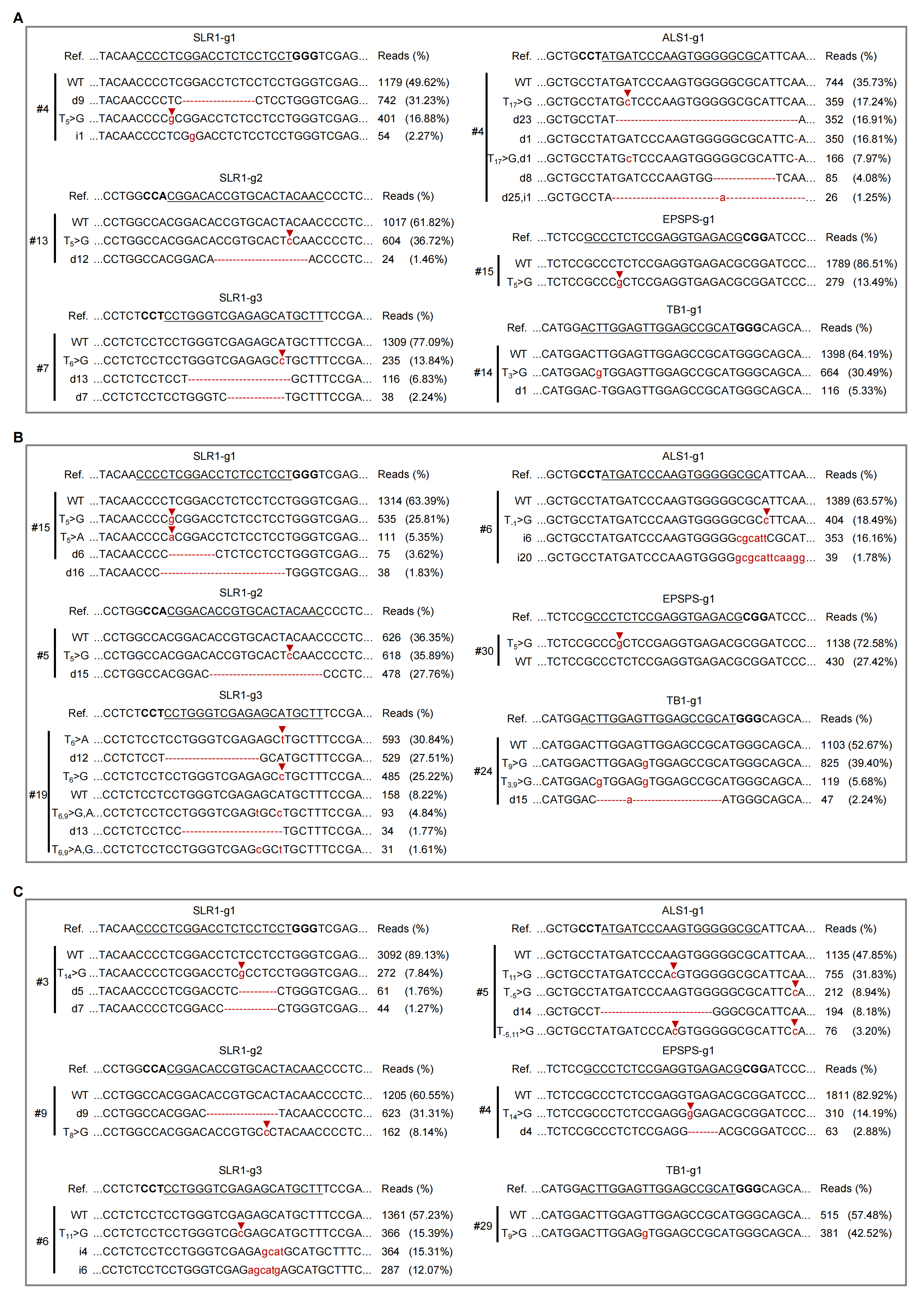


**Supplementary figure 4. Representative amplicon sequencing results of selected T_0_ plants.** **(A-C)** T_0_ plants were subjected to Hi-TOM sequencing with an 1% filter threshold. The mutation types and positions of each sample are listed in detail, including reads number, ratio, mutation types, mutation bases and DNA sequences. Data shown the genotyping results of representative T_0_ plant edited by CE_1029_TDG3 (A), CE_1046_TDG3 (B), and CE_1249_TDG3 (C). The PAMs are marked in bold, the protospacers are underlined, mutations are highlighted in red and the base substitutions are denoted by red triangles.


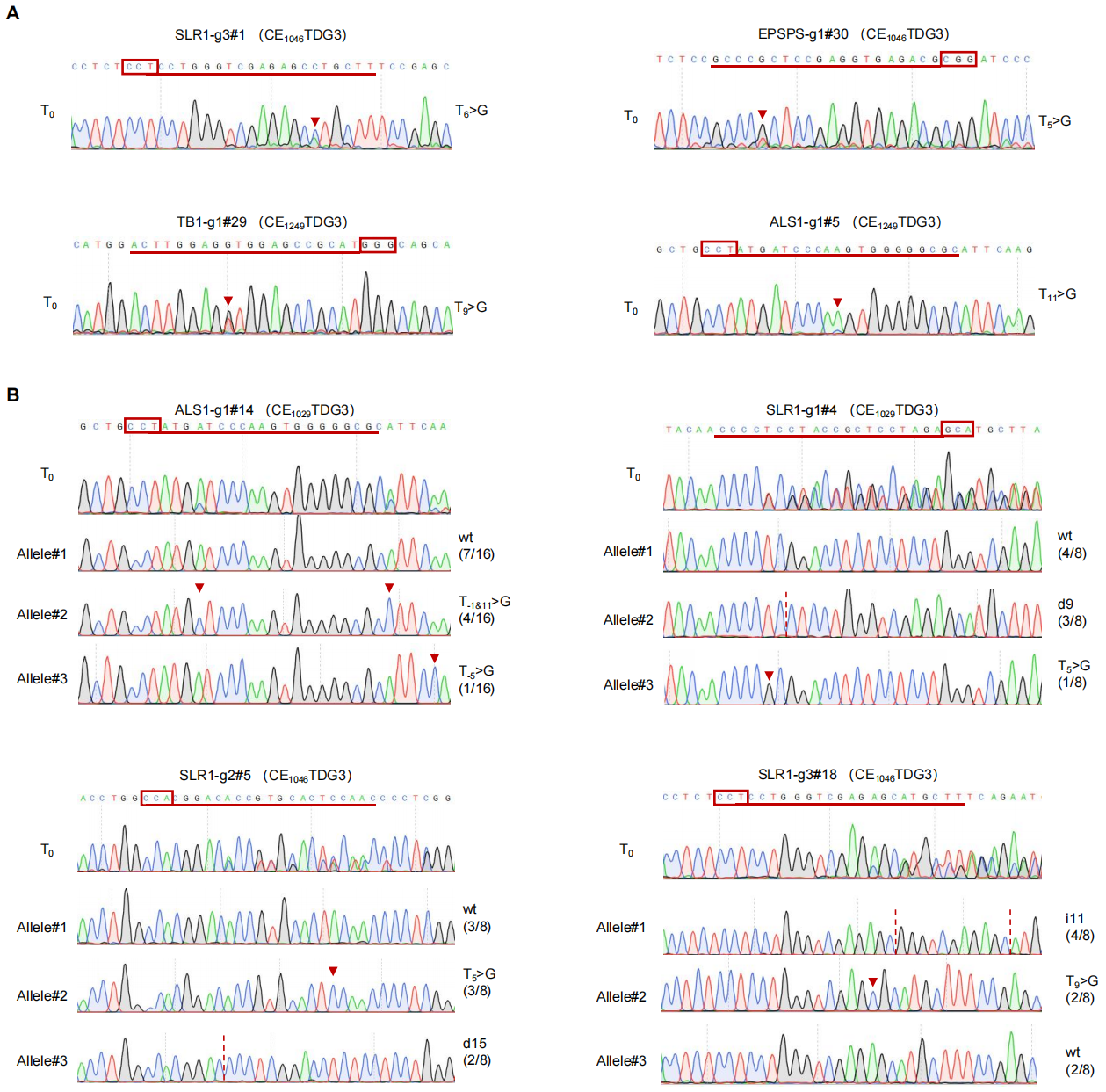


**Supplementary figure 5. Representative subcloning sequencing results of selected T_0_ plants.** **(A-C)** T_0_ plants with chimerism > 10% are subjected to Sanger sequencing and subcloning sequencing. Data shown the Sanger sequencing and subcloning sequencing results of representative T_0_ plant. The PAMs are marked by rectangles, and the protospacers are underlined. The base substitutions are marked by red triangles and the indels are marked by red dashed lines.


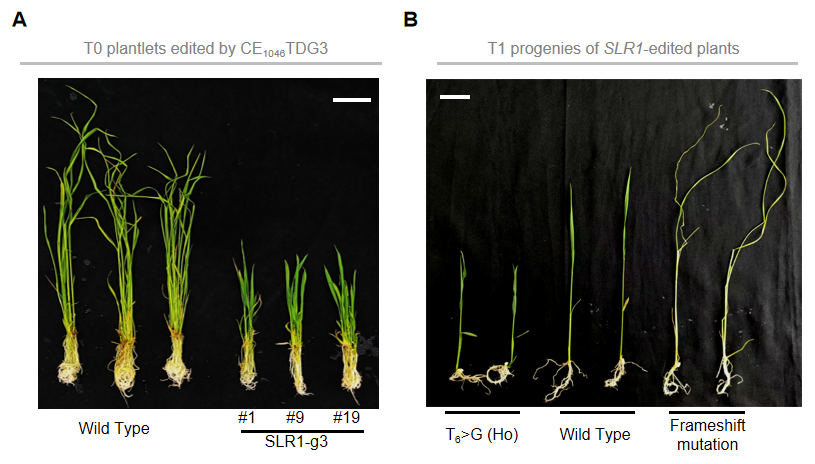


**Supplementary figure 6.** Representative phenotype of selected plants. **(A-B)** Phenotype of T_0_ (A) and T_1_ (B) rice plants with editing on SLR1-g3; scale bar, 3 cm.

**Supplementary sequence 1.** Sequences of related vectors and genes.

**>OsU6::gRNA::polyT** (the OsU6 promoter is marked in yellow, the gRNA scaffold is marked in red, and the polyT terminal signal is marked in blue.)

ggatcatgaaccaacggcctggctgtatttggtggttgtgtagggagatggggagaagaaaagcccgattctcttcgctgtgatgggctggatgcatgcgggggagcgggaggcccaagtacgtgcacggtgagcggcccacagggcgagtgtgagcgcgagaggcgggaggaacagtttagtaccacattgcccagctaactcgaacgcgaccaacttataaacccgcgcgctgtcgcttgtgagagacctcggtctccgttttagagctagaaatagcaagttaaaataaggctagtccgttatcaacttgaaaaagtggcaccgagtcggtgcttttttt

**>Amino acid sequence of CE_1046_hUNG** (the Cas9 nickase is marked in yellow, the Cas-embedded hUNG is marked in red, the N-terminal NLS peptide is marked in blue, and the C-terminal VirD2 NLS is marked in green.)

MKRTADGSEFESPKKKRKVSGGSDKKYSIGLAIGTNSVGWAVITDEYKVPSKKFKVLGNTDRHSIKKNLIGALLFDSGETAEATRLKRTARRRYTRRKNRICYLQEIFSNEMAKVDDSFFHRLEESFLVEEDKKHERHPIFGNIVDEVAYHEKYPTIYHLRKKLVDSTDKADLRLIYLALAHMIKFRGHFLIEGDLNPDNSDVDKLFIQLVQTYNQLFEENPINASGVDAKAILSARLSKSRRLENLIAQLPGEKKNGLFGNLIALSLGLTPNFKSNFDLAEDAKLQLSKDTYDDDLDNLLAQIGDQYADLFLAAKNLSDAILLSDILRVNTEITKAPLSASMIKRYDEHHQDLTLLKALVRQQLPEKYKEIFFDQSKNGYAGYIDGGASQEEFYKFIKPILEKMDGTEELLVKLNREDLLRKQRTFDNGSIPHQIHLGELHAILRRQEDFYPFLKDNREKIEKILTFRIPYYVGPLARGNSRFAWMTRKSEETITPWNFEEVVDKGASAQSFIERMTNFDKNLPNEKVLPKHSLLYEYFTVYNELTKVKYVTEGMRKPAFLSGEQKKAIVDLLFKTNRKVTVKQLKEDYFKKIECFDSVEISGVEDRFNASLGTYHDLLKIIKDKDFLDNEENEDILEDIVLTLTLFEDREMIEERLKTYAHLFDDKVMKQLKRRRYTGWGRLSRKLINGIRDKQSGKTILDFLKSDGFANRNFMQLIHDDSLTFKEDIQKAQVSGQGDSLHEHIANLAGSPAIKKGILQTVKVVDELVKVMGRHKPENIVIEMARENQTTQKGQKNSRERMKRIEEGIKELGSQILKEHPVENTQLQNEKLYLYYLQNGRDMYVDQELDINRLSDYDVDHIVPQSFLKDDSIDNKVLTRSDKNRGKSDNVPSEEVVKKMKNYWRQLLNAKLITQRKFDNLTKAERGGLSELDKAGFIKRQLVETRQITKHVAQILDSRMNTKYDENDKLIREVKVITLKSKLVSDFRKDFQFYKVREINNYHHAHDAYLNAVVGTALIKKYPKLESEFVYGDYKVYDVRKMIAKSEQEIGKATAKYFFYSNIMNFFGGGGSGVFCLGPWGLGRKLRTPGKGPLQLLSRLCGDHLQAIPAKKAPAGQEEPGTPPSSPLSAEQLDRIQRNKAAALLRLAARNVPVGFGESWKKHLSGEFGKPYFIKLMGFVAEERKHYTVYPPPHQVFTWTQMCDIKDVKVVILGQDPYHGPNQAHGLCFSVQRPVPPPPSLENIYKELSTDIEDFVHPGHGDLSGWAKQGVLLLNAVLTVRAHQANSHKERGWEQFTDAVVSWLNQNSNGLVFLLWGSYAQKKGSAIDRKRHHVLQTAHPSPLSVYRGFFGCRHFSKTNELLQKSGKKPIDWKELGGGGSIETNGETGEIVWDKGRDFATVRKVLSMPQVNIVKKTEVQTGGFSKESILPKRNSDKLIARKKDWDPKKYGGFDSPTVAYSVLVVAKVEKGKSKKLKSVKELLGITIMERSSFEKNPIDFLEAKGYKEVKKDLIIKLPKYSLFELENGRKRMLASAGELQKGNELALPSKYVNFLYLASHYEKLKGSPEDNEQKQLFVEQHKHYLDEIIEQISEFSKRVILADANLDKVLSAYNKHRDKPIREQAENIIHLFTLTNLGAPAAFKYFDTTIDRKRYTSTKEVLDATLIHQSITGLYETRIDLSQLGGDTSSGGSASPKRPRDRHDGELGGRKRARG

**>hTDG** (Y147A is marked in black)

GVFCLGPWGLGRKLRTPGKGPLQLLSRLCGDHLQAIPAKKAPAGQEEPGTPPSSPLSAEQLDRIQRNKAAALLRLAARNVPVGFGESWKKHLSGEFGKPYFIKLMGFVAEERKHYTVYPPPHQVFTWTQMCDIKDVKVVILGQDP**A**HGPNQAHGLCFSVQRPVPPPPSLENIYKELSTDIEDFVHPGHGDLSGWAKQGVLLLNAVLTVRAHQANSHKERGWEQFTDAVVSWLNQNSNGLVFLLWGSYAQKKGSAIDRKRHHVLQTAHPSPLSVYRGFFGCRHFSKTNELLQKSGKKPIDWKEL

**>TDG-EK** (G107E, Y147A, and R260K is marked in black)

GVFCLGPWGLGRKLRTPGKGPLQLLSRLCGDHLQAIPAKKAPAGQEEPGTPPSSPLSAEQLDRIQRNKAAALLRLAARNVPVGFGESWKKHLSGEFGKPYFIKLM**E**FVAEERKHYTVYPPPHQVFTWTQMCDIKDVKVVILGQDP**A**HGPNQAHGLCFSVQRPVPPPPSLENIYKELSTDIEDFVHPGHGDLSGWAKQGVLLLNAVLTVRAHQANSHKERGWEQFTDAVVSWLNQNSNGLVFLLWGSYAQKKGSAIDRK**K**HHVLQTAHPSPLSVYRGFFGCRHFSKTNELLQKSGKKPIDWKEL

**>TDG3** (F85L, G107R, L142V, Y147A, K175E, S237L, A255V, K259E, T266A, V274A, Y275S is marked in black)

GVFCLGPWGLGRKLRTPGKGPLQLLSRLCGDHLQAIPAKKAPAGQEEPGTPPSSPLSAEQLDRIQRNKAAALLRLAARNVPVG**L**GESWKKHLSGEFGKPYFIKLM**R**FVAEERKHYTVYPPPHQVFTWTQMCDIKDVKVVI**V**GQDP**A**HGPNQAHGLCFSVQRPVPPPPSLENIY**E**ELSTDIEDFVHPGHGDLSGWAKQGVLLLNAVLTVRAHQANSHKERGWEQFTDAVVSWLNQN**L**NGLVFLLWGSYAQKKGS**V**IDR**E**RHHVLQ**A**AHPSPLS**AS**RGFFGCRHFSKTNELLQKSGKKPIDWKEL

**Supplementary sequence 2.** DNA sequence of the NLuc cassette of pDLuc-TBE.

**>2x35S::mNLuc::PolyA** (the 2x35S promoter is marked in yellow, the mutated NLuc gene is marked in red, the GGA>TGA mutation are marked bold, and the NOS terminator is marked in blue.)

AGCTTGCATGCCTGCAGGTCCCCAGATTAGCCTTTTCAATTTCAGAAAGAATGCTAACCCACAGATGGTTAGAGAGGCTTACGCAGCAGGTCTCATCAAGACGATCTACCCGAGCAATAATCTCCAGGAAATCAAATACCTTCCCAAGAAGGTTAAAGATGCAGTCAAAAGATTCAGGACTAACTGCATCAAGAACACAGAGAAAGATATATTTCTCAAGATCAGAAGTACTATTCCAGTATGGACGATTCAAGGCTTGCTTCACAAACCAAGGCAAGTAATAGAGATTGGAGTCTCTAAAAAGGTAGTTCCCACTGAATCAAAGGCCATGGAGTCAAAGATTCAAATAGAGGACCTAACAGAACTCGCCGTAAAGACTGGCGAACAGTTCATACAGAGTCTCTTACGACTCAATGACAAGAAGAAAATCTTCGTCAACATGGTGGAGCACGACACACTTGTCTACTCCAAAAATATCAAAGATACAGTCTCAGAAGACCAAAGGGCAATTGAGACTTTTCAACAAAGGGTAATATCCGGAAACCTCCTCGGATTCCATTGCCCAGCTATCTGTCACTTTATTGTGAAGATAGTGGAAAAGGAAGGTGGCTCCTACAAATGCCATCATTGCGATAAAGGAAAGGCCATCGTTGAAGATGCCTCTGCCGACAGTGGTCCCAAAGATGGACCCCCACCCACGAGGAGCATCGTGGAAAAAGAAGACGTTCCAACCACGTCTTCAAAGCAAGTGGATTGATGTGATATCTCCACTGACGTAAGGGATGACGCACAATCCCACTATCCTTCGCAAGACCCTTCCTCTATATAAGGAAGTTCATTTCATTTGGAGAGAACACGGGGGACAAGCTTGGTACCTCTAGAATGGTCTTCACACTCGAAGATTTCGTTGGGGACTGGCGACAGACAGCCGGCTACAACCTGGACCAAGTCCTTGAACAGGGAGGTGTGTCCAGTTTGTTTCAGAATCTCGGGGTGTCCGTAACTCCGATCCAAAGGATTGTCCTGAGCGGTGAAAATGGGCTGAAGATCGACATCCATGTCATCATCCCGTATGAAGGACTGAGC**TGA**GACCAAATGGGGCAGATCGAAAAAATTTTTAAGGTGGTGTACCCTGTGGATGATCATCACTTTAAGGTGATCCTGCACTATGGCACACTGGTAATCGACGGGGTTACGCCGAACATGATCGACTATTTCGGACGGCCGTATGAAGGCATCGCCGTGTTCGACGGCAAAAAGATCACTGTAACAGGGACCCTGTGGAACGGCAACAAAATTATCGACGAGCGCCTGATCAACCCCGACGGCTCCCTGCTGTTCCGAGTAACCATCAACGGAGTGACCGGCTGGCGGCTGTGCGAACGCATTCTGGCGTAAGGATCCCTCGAGCTCCTGCAGATCGTTCAAACATTTGGCAATAAAGTTTCTTAAGATTGAATCCTGTTGCCGGTCTTGCGATGATTATCATATAATTTCTGTTGAATTACGTTAAGCATGTAATAATTAACATGTAATGCATGACGTTATTTATGAGATGGGTTTTTATGATTAGAGTCCCGCAATTATACATTTAATACGCGATAGAAAACAAAATATAGCGCGCAAACTAGGATAAATTATCGCGCGCGGTGTCATCTATGTTACTAGATCGGT

**Supplementary table 1.** Summary of the targeted sites.

| **Name** | **Target sequence (5'-3')** | **Locus** | **Region** | **Experiments** |
| --- | --- | --- | --- | --- |
| sgRNA-T9 | GACTGAGCTGAGACCAAATG**GGG** | *NLuc* | CDS | Recovery of NLuc reporter gene |
| sgRNA-T10 | GGACTGAGCTGAGACCAAAT**GGG** | *NLuc* | CDS |  |
| sgRNA-T11 | AGGACTGAGCTGAGACCAAA**TGG** | *NLuc* | CDS |  |
| SLR1-g1 | CCCCTCGGACCTCTCCTCCT**GGG** | *SLR1* | CDS | Genome editing at six endogenous loci |
| SLR1-g2 | GTTGTAGTGCACGGTGTCCG**TGG** |  | CDS |  |
| SLR1-g3 | AAGCATGCTCTCGACCCAGG**AGG** |  | CDS |  |
| ALS1-g1 | GCGCCCCCACTTGGGATCAT**AGG** | *OsALS1* | CDS |  |
| EPSPS-g1 | GCCCTCTCCGAGGTGAGACG**CGG** | *OsEPSPS* | CDS |  |
| TB1-g1 | ACTTGGAGTTGGAGCCGCAT**GGG** | *OsTB1* | CDS |  |

**Note:** The PAMs are marked in bold.

**Supplementary table 2.** Summary of CE-TBEs induced editing in rice T_0_ plantlets.

| **Editors** | **target** | **T0 plants** | **Plant with InDels** | **Plant with base edits** | | |
| --- | --- | --- | --- | --- | --- | --- |
|  |  |  |  | **T-to-G** | **T-to-A** | **T-to-C** |
| CE_1029_hTDG | SLR1-g1 | 29 | 0 | 0 | 0 | 0 |
|  | SLR1-g2 | 31 | 0 | 0 | 0 | 0 |
|  | SLR1-g3 | 24 | 0 | 0 | 0 | 0 |
|  | ALS1-g1 | 28 | 0 | 0 | 0 | 0 |
|  | EPSPS-g1 | 25 | 0 | 0 | 0 | 0 |
|  | TB1-g1 | 36 | 0 | 0 | 0 | 0 |
| CE_1029_TDG-EK | SLR1-g1 | 22 | 0 | 0 | 0 | 0 |
|  | SLR1-g2 | 21 | 0 | 0 | 0 | 0 |
|  | SLR1-g3 | 31 | 0 | 0 | 0 | 0 |
|  | ALS1-g1 | 27 | 0 | 0 | 0 | 0 |
|  | EPSPS-g1 | 22 | 0 | 0 | 0 | 0 |
|  | TB1-g1 | 25 | 0 | 0 | 0 | 0 |
| **CE_1029_TDG3** | SLR1-g1 | 25 | 3 (12%) | 2 (8%) | 0 | 0 |
|  | SLR1-g2 | 33 | 10 (30.3%) | 1 (3%) | 0 | 0 |
|  | SLR1-g3 | 18 | 0 | 2 (11.1%) | 0 | 0 |
|  | ALS1-g1 | 32 | 5 (15.6%) | 10 (31.3%) | 0 | 0 |
|  | EPSPS-g1 | 20 | 0 | 1 (5%) | 0 | 0 |
|  | TB1-g1 | 24 | 0 | 2 (8.3%) | 0 | 0 |
| CE_1046_hTDG | SLR1-g1 | 28 | 0 | 0 | 0 | 0 |
|  | SLR1-g2 | 26 | 1 (3.8%) | 0 | 0 | 0 |
|  | SLR1-g3 | 77 | 0 | 0 | 0 | 0 |
|  | ALS1-g1 | 31 | 0 | 0 | 0 | 0 |
|  | EPSPS-g1 | 65 | 1 (1.5%) | 0 | 0 | 0 |
|  | TB1-g1 | 35 | 0 | 0 | 0 | 0 |
| CE_1046_TDG-EK | SLR1-g1 | 55 | 5 (9.1%) | 0 | 0 | 0 |
|  | SLR1-g2 | 38 | 2 (5.3%) | 0 | 0 | 0 |
|  | SLR1-g3 | 28 | 0 | 1 (3.6%) | 0 | 0 |
|  | ALS1-g1 | 33 | 2 (6.1%) | 0 | 0 | 0 |
|  | EPSPS-g1 | 32 | 0 | 0 | 0 | 0 |
|  | TB1-g1 | 48 | 1 (2.1%) | 1 (2.1%) | 0 | 0 |
| **CE_1046_TDG3** | SLR1-g1 | 31 | 8 (25.8%) | 1 (3.2%) | 0 | 0 |
|  | SLR1-g2 | 23 | 9 (39.1%) | 3 (13%) | 0 | 0 |
|  | SLR1-g3 | 34 | 13 (38.2%) | 13 (38.2%) | 2 (5.9%) | 0 |
|  | ALS1-g1 | 11 | 2 (18.2%) | 1 (9.1%) | 0 | 0 |
|  | EPSPS-g1 | 35 | 12 (34.3%) | 4 (11.4%) | 0 | 0 |
|  | TB1-g1 | 31 | 4 (12.9%) | 5 (16.1%) | 0 | 0 |
| CE_1249_hTDG | SLR1-g1 | 26 | 0 | 0 | 0 | 0 |
|  | SLR1-g2 | 37 | 0 | 0 | 0 | 0 |
|  | SLR1-g3 | 29 | 0 | 0 | 0 | 0 |
|  | ALS1-g1 | 21 | 0 | 0 | 0 | 0 |
|  | EPSPS-g1 | 26 | 0 | 0 | 0 | 0 |
|  | TB1-g1 | 22 | 0 | 0 | 0 | 0 |
| CE_1249_TDG-EK | SLR1-g1 | 42 | 0 | 0 | 0 | 0 |
|  | SLR1-g2 | 33 | 0 | 0 | 0 | 0 |
|  | SLR1-g3 | 30 | 1 (3.3%) | 0 | 0 | 0 |
|  | ALS1-g1 | 58 | 0 | 0 | 0 | 0 |
|  | EPSPS-g1 | 25 | 0 | 0 | 0 | 0 |
|  | TB1-g1 | 53 | 0 | 0 | 0 | 0 |
| **CE_1249_TDG3** | SLR1-g1 | 35 | 0 | 0 | 0 | 0 |
|  | SLR1-g2 | 15 | 1 (6.7%) | 0 | 0 | 0 |
|  | SLR1-g3 | 23 | 2 (8.7%) | 2 (8.7%) | 0 | 0 |
|  | ALS1-g1 | 17 | 3 (17.6%) | 1 (5.9%) | 0 | 0 |
|  | EPSPS-g1 | 23 | 1 (4.3%) | 1 (4.3%) | 0 | 0 |
|  | TB1-g1 | 32 | 0 | 2 (6.3%) | 0 | 0 |

**Note:** The mutations are determined with a 10% HiTOM threshold. The base edited alleles only include the pure base conversions without indels. CE-TDG3 constructs are marked in red.

**Supplementary table 3.** Summary of HiTOM sequencing results of T_0_ transgenic plants.

**Note:** Data shown the target positions with valid editing (Chimerism > 10%).

| **CE_1046_TDG-EK** | | | **SLR1-g3** | | |
| --- | --- | --- | --- | --- | --- |
| Total  T0 plants | Genotyping method | InDel | T11 | | |
|  |  |  | T-to-G | T-to-C | T-to-A |
| 28 | Reads%>10% | 0 | 1 | 0 | 0 |
| #1 | HiTOM | 0.00% | 0.00% | 0.00% | 0.00% |
| #2 | HiTOM | 0.00% | 0.00% | 0.00% | 0.00% |
| #3 | HiTOM | 1.14% | 0.00% | 0.00% | 0.00% |
| #4 | HiTOM | 0.00% | 0.00% | 0.00% | 0.00% |
| #5 | HiTOM | 0.00% | 1.43% | 0.00% | 0.00% |
| #6 | HiTOM | 0.00% | 10.89% | 0.00% | 0.00% |
| #7 | HiTOM | 0.00% | 0.00% | 0.00% | 0.00% |
| #8 | HiTOM | 0.00% | 0.00% | 0.00% | 0.00% |
| #9 | HiTOM | 0.00% | 0.00% | 0.00% | 0.00% |
| #10 | HiTOM | 0.00% | 0.00% | 0.00% | 0.00% |
| #11 | HiTOM | 0.00% | 0.00% | 0.00% | 0.00% |
| #12 | HiTOM | 0.00% | 0.00% | 0.00% | 0.00% |
| #13 | HiTOM | 0.00% | 0.00% | 0.00% | 0.00% |
| #14 | HiTOM | 0.00% | 0.00% | 0.00% | 0.00% |
| #15 | HiTOM | 0.00% | 0.00% | 0.00% | 0.00% |
| #16 | HiTOM | 0.00% | 0.00% | 0.00% | 0.00% |
| #17 | HiTOM | 0.00% | 0.00% | 0.00% | 0.00% |
| #18 | HiTOM | 0.00% | 0.00% | 0.00% | 0.00% |
| #19 | HiTOM | 0.00% | 0.00% | 0.00% | 0.00% |
| #20 | HiTOM | 0.00% | 0.00% | 0.00% | 0.00% |
| #21 | HiTOM | 0.00% | 0.00% | 0.00% | 0.00% |
| #22 | HiTOM | 0.00% | 0.00% | 0.00% | 0.00% |
| #23 | HiTOM | 0.00% | 0.00% | 0.00% | 0.00% |
| #24 | HiTOM | 0.00% | 0.00% | 0.00% | 0.00% |
| #25 | HiTOM | 0.00% | 0.00% | 0.00% | 0.00% |
| #26 | HiTOM | 2.40% | 0.00% | 0.00% | 0.00% |
| #27 | HiTOM | 0.00% | 0.00% | 0.00% | 0.00% |
| #28 | HiTOM | 0.00% | 0.00% | 0.00% | 0.00% |

**Supplementary table 3.** Continued.

| **CE_1046_TDG-EK** | | | **TB1-g1** | | |
| --- | --- | --- | --- | --- | --- |
| Total  T0 plants | Genotyping method | InDel | T3 | | |
|  |  |  | T-to-G | T-to-C | T-to-A |
| 48 | Reads%>10% | 1 | 1 | 0 | 0 |
| #1 | HiTOM | 0.00% | 0.00% | 0.00% | 0.00% |
| #2 | HiTOM | 0.00% | 0.00% | 0.00% | 0.00% |
| #3 | HiTOM | 0.00% | 0.00% | 0.00% | 0.00% |
| #4 | HiTOM | 0.00% | 0.00% | 0.00% | 0.00% |
| #5 | HiTOM | 0.00% | 0.00% | 0.00% | 0.00% |
| #6 | HiTOM | 0.00% | 0.00% | 0.00% | 0.00% |
| #7 | HiTOM | 0.00% | 0.00% | 0.00% | 0.00% |
| #8 | HiTOM | 0.00% | 0.00% | 0.00% | 0.00% |
| #9 | HiTOM | 0.00% | 0.00% | 0.00% | 0.00% |
| #10 | HiTOM | 0.00% | 0.00% | 0.00% | 0.00% |
| #11 | HiTOM | 0.00% | 0.00% | 0.00% | 0.00% |
| #12 | HiTOM | 0.00% | 0.00% | 0.00% | 0.00% |
| #13 | HiTOM | 0.00% | 0.00% | 0.00% | 0.00% |
| #14 | HiTOM | 0.00% | 0.00% | 0.00% | 0.00% |
| #15 | HiTOM | 0.00% | 0.00% | 0.00% | 0.00% |
| #16 | HiTOM | 0.00% | 0.00% | 0.00% | 0.00% |
| #17 | HiTOM | 0.00% | 0.00% | 0.00% | 0.00% |
| #18 | HiTOM | 0.00% | 0.00% | 0.00% | 0.00% |
| #19 | HiTOM | 0.00% | 0.00% | 0.00% | 0.00% |
| #20 | HiTOM | 0.00% | 0.00% | 0.00% | 0.00% |
| #21 | HiTOM | 0.00% | 0.00% | 0.00% | 0.00% |
| #22 | HiTOM | 0.00% | 0.00% | 0.00% | 0.00% |
| #23 | HiTOM | 0.00% | 0.00% | 0.00% | 0.00% |
| #24 | HiTOM | 0.00% | 0.00% | 0.00% | 0.00% |
| #25 | HiTOM | 0.00% | 0.00% | 0.00% | 0.00% |
| #26 | HiTOM | 0.00% | 0.00% | 0.00% | 0.00% |
| #27 | HiTOM | 0.00% | 0.00% | 0.00% | 0.00% |
| #28 | HiTOM | 0.00% | 0.00% | 0.00% | 0.00% |
| #29 | HiTOM | 0.00% | 0.00% | 0.00% | 0.00% |
| #30 | HiTOM | 0.00% | 0.00% | 0.00% | 0.00% |
| #31 | HiTOM | 0.00% | 0.00% | 0.00% | 0.00% |
| #32 | HiTOM | 0.00% | 0.00% | 0.00% | 0.00% |
| #33 | HiTOM | 0.00% | 0.00% | 0.00% | 0.00% |
| #34 | HiTOM | 5.35% | 13.20% | 0.00% | 0.00% |
| #35 | HiTOM | 0.00% | 0.00% | 0.00% | 0.00% |
| #36 | HiTOM | 0.00% | 0.00% | 0.00% | 0.00% |
| #37 | HiTOM | 0.00% | 0.00% | 0.00% | 0.00% |
| #38 | HiTOM | 0.00% | 0.00% | 0.00% | 0.00% |
| #39 | HiTOM | 0.00% | 0.00% | 0.00% | 0.00% |
| #40 | HiTOM | 0.00% | 3.22% | 0.00% | 0.00% |
| #41 | HiTOM | 0.00% | 0.00% | 0.00% | 0.00% |
| #42 | HiTOM | 0.00% | 0.00% | 0.00% | 0.00% |
| #43 | HiTOM | 0.00% | 0.00% | 0.00% | 0.00% |
| #44 | HiTOM | 0.00% | 0.00% | 0.00% | 0.00% |
| #45 | HiTOM | 0.00% | 0.00% | 0.00% | 0.00% |
| #46 | HiTOM | 17.32% | 0.00% | 0.00% | 0.00% |
| #47 | HiTOM | 0.00% | 0.00% | 0.00% | 0.00% |
| #48 | HiTOM | 0.00% | 0.00% | 0.00% | 0.00% |

**Supplementary table 3.** Continued.

| **CE_1029_TDG3** | | | **SLR1-g1** | | |
| --- | --- | --- | --- | --- | --- |
| Total  T0 plants | Genotyping method | InDel | T5 | | |
|  |  |  | T-to-G | T-to-C | T-to-A |
| 25 | Reads%>10% | 3 | 2 | 0 | 0 |
| #1 | HiTOM | 0.00% | 0.00% | 0.00% | 0.00% |
| #2 | HiTOM | 8.33% | 12.95% | 0.00% | 0.00% |
| #3 | HiTOM | 0.00% | 0.00% | 0.00% | 0.00% |
| #4 | HiTOM | 33.50% | 16.88% | 0.00% | 0.00% |
| #5 | HiTOM | 0.00% | 0.00% | 0.00% | 0.00% |
| #6 | HiTOM | 0.00% | 0.00% | 0.00% | 0.00% |
| #7 | HiTOM | 19.86% | 8.99% | 0.00% | 0.00% |
| #8 | HiTOM | 0.00% | 0.00% | 0.00% | 0.00% |
| #9 | HiTOM | 0.00% | 0.00% | 0.00% | 0.00% |
| #10 | HiTOM | 27.34% | 0.00% | 1.84% | 0.00% |
| #11 | HiTOM | 0.00% | 0.00% | 0.00% | 0.00% |
| #12 | HiTOM | 5.33% | 2.47% | 0.00% | 0.00% |
| #13 | HiTOM | 0.00% | 1.35% | 0.00% | 0.00% |
| #14 | HiTOM | 7.27% | 0.00% | 0.00% | 0.00% |
| #15 | HiTOM | 0.00% | 0.00% | 0.00% | 0.00% |
| #16 | HiTOM | 2.41% | 0.00% | 0.00% | 0.00% |
| #17 | HiTOM | 0.00% | 0.00% | 0.00% | 0.00% |
| #18 | HiTOM | 0.00% | 0.00% | 0.00% | 0.00% |
| #19 | HiTOM | 0.00% | 0.00% | 3.08% | 0.00% |
| #20 | HiTOM | 0.00% | 0.00% | 0.00% | 0.00% |
| #21 | HiTOM | 0.00% | 0.00% | 0.00% | 0.00% |
| #22 | HiTOM | 0.00% | 0.00% | 0.00% | 0.00% |
| #23 | HiTOM | 0.00% | 0.00% | 2.74% | 0.00% |
| #24 | HiTOM | 0.00% | 0.00% | 0.00% | 0.00% |
| #25 | HiTOM | 0.00% | 0.00% | 0.00% | 0.00% |

**Supplementary table 3.** Continued.

| **CE_1029_TDG3** | | | **SLR1-g2** | | |
| --- | --- | --- | --- | --- | --- |
| Total  T0 plants | Genotyping method | InDel | T5 | | |
|  |  |  | T-to-G | T-to-C | T-to-A |
| 33 | Reads%>10% | 10 | 1 | 0 | 0 |
| #1 | HiTOM | 0.00% | 0.00% | 0.00% | 0.00% |
| #2 | HiTOM | 39.61% | 0.00% | 0.00% | 0.00% |
| #3 | HiTOM | 0.00% | 0.00% | 0.00% | 0.00% |
| #4 | HiTOM | 14.81% | 0.00% | 0.00% | 0.00% |
| #5 | HiTOM | 13.82% | 0.00% | 0.00% | 0.00% |
| #6 | HiTOM | 0.00% | 0.00% | 0.00% | 0.00% |
| #7 | HiTOM | 0.00% | 0.00% | 0.00% | 0.00% |
| #8 | HiTOM | 0.00% | 0.00% | 0.00% | 0.00% |
| #9 | HiTOM | 3.77% | 3.56% | 0.00% | 0.00% |
| #10 | HiTOM | 0.00% | 0.00% | 0.00% | 0.00% |
| #11 | HiTOM | 0.00% | 1.57% | 0.00% | 0.00% |
| #12 | HiTOM | 9.18% | 4.56% | 0.00% | 0.00% |
| #13 | HiTOM | 1.46% | 36.72% | 0.00% | 0.00% |
| #14 | HiTOM | 0.00% | 0.00% | 0.00% | 0.00% |
| #15 | HiTOM | 18.37% | 0.00% | 0.00% | 0.00% |
| #16 | HiTOM | 14.04% | 1.05% | 0.00% | 0.00% |
| #17 | HiTOM | 0.00% | 0.00% | 0.00% | 0.00% |
| #18 | HiTOM | 0.00% | 0.00% | 0.00% | 0.00% |
| #19 | HiTOM | 0.00% | 0.00% | 0.00% | 0.00% |
| #20 | HiTOM | 10.04% | 0.00% | 0.00% | 0.00% |
| #21 | HiTOM | 1.51% | 0.00% | 0.00% | 0.00% |
| #22 | HiTOM | 0.00% | 0.00% | 0.00% | 0.00% |
| #23 | HiTOM | 0.00% | 0.00% | 0.00% | 0.00% |
| #24 | HiTOM | 0.00% | 0.00% | 0.00% | 0.00% |
| #25 | HiTOM | 0.00% | 0.00% | 0.00% | 0.00% |
| #26 | HiTOM | 0.00% | 0.00% | 0.00% | 0.00% |
| #27 | HiTOM | 33.35% | 1.59% | 0.00% | 0.00% |
| #28 | HiTOM | 10.65% | 5.15% | 0.00% | 0.00% |
| #29 | HiTOM | 0.00% | 0.00% | 0.00% | 0.00% |
| #30 | HiTOM | 0.00% | 0.00% | 0.00% | 0.00% |
| #31 | HiTOM | 15.03% | 8.29% | 1.22% | 0.00% |
| #32 | HiTOM | 0.00% | 0.00% | 0.00% | 0.00% |
| #33 | HiTOM | 38.43% | 0.00% | 0.00% | 0.00% |

**Supplementary table 3.** Continued.

| **CE_1029_TDG3** | | | **SLR1-g3** | | |
| --- | --- | --- | --- | --- | --- |
| Total  T0 plants | Genotyping method | InDel | T6 | | |
|  |  |  | T-to-G | T-to-C | T-to-A |
| 22 | Reads%>10% | 0 | 2 | 0 | 0 |
| #1 | HiTOM | 0.00% | 0.00% | 0.00% | 0.00% |
| #2 | HiTOM | 8.57% | 7.56% | 0.00% | 0.00% |
| #3 | HiTOM | 0.00% | 0.00% | 0.00% | 0.00% |
| #4 | HiTOM | 5.35% | 0.00% | 0.00% | 0.00% |
| #5 | HiTOM | 0.00% | 1.78% | 0.00% | 0.00% |
| #6 | HiTOM | 0.00% | 0.00% | 0.00% | 0.00% |
| #7 | HiTOM | 9.07% | 13.84% | 0.00% | 0.00% |
| #8 | HiTOM | 1.28% | 0.00% | 0.00% | 1.87% |
| #9 | HiTOM | 0.00% | 3.01% | 0.00% | 0.00% |
| #10 | HiTOM | 0.00% | 0.00% | 0.00% | 0.00% |
| #11 | HiTOM | 0.00% | 0.00% | 0.00% | 0.00% |
| #12 | HiTOM | 0.00% | 0.00% | 0.00% | 0.00% |
| #13 | HiTOM | 9.31% | 16.26% | 0.00% | 0.00% |
| #14 | HiTOM | 0.00% | 0.00% | 0.00% | 0.00% |
| #15 | HiTOM | 0.00% | 0.00% | 0.00% | 0.00% |
| #16 | HiTOM | 0.00% | 5.36% | 0.00% | 0.00% |
| #17 | HiTOM | 0.00% | 0.00% | 0.00% | 0.00% |
| #18 | HiTOM | 2.16% | 3.40% | 0.00% | 0.00% |

**Supplementary table 3.** Continued.

| **CE_1029_TDG3** | | | **ALS1-g1** | | | | | | | | | | | | | | | | | |
| --- | --- | --- | --- | --- | --- | --- | --- | --- | --- | --- | --- | --- | --- | --- | --- | --- | --- | --- | --- | --- |
| Total  T0 plants | Genotyping method | InDel | T-6 | | | T-5 | | | A-2 | | | T-1 | | | T11 | | | T17 | | |
|  |  |  | T-to-G | T-to-C | T-to-A | T-to-G | T-to-C | T-to-A | A-to-C | A-to-G | A-to-T | T-to-G | T-to-C | T-to-A | T-to-G | T-to-C | T-to-A | T-to-G | T-to-C | T-to-A |
| 32 | Reads%>10% | 5 | 1 | 0 | 0 | 1 | 0 | 0 | 2 | 0 | 0 | 6 | 0 | 0 | 3 | 0 | 0 | 1 | 0 | 0 |
| #1 | HiTOM | 0.00% | 0.00% | 0.00% | 0.00% | 0.00% | 0.00% | 0.00% | 0.00% | 0.00% | 0.00% | 3.93% | 0.00% | 0.00% | 1.19% | 0.00% | 0.00% | 0.00% | 0.00% | 0.00% |
| #2 | HiTOM | 0.00% | 0.00% | 0.00% | 0.00% | 0.00% | 0.00% | 0.00% | 0.00% | 0.00% | 0.00% | 11.85% | 0.00% | 0.00% | 0.00% | 0.00% | 0.00% | 0.00% | 0.00% | 0.00% |
| #3 | HiTOM | 0.00% | 0.00% | 0.00% | 0.00% | 0.00% | 0.00% | 0.00% | 0.00% | 0.00% | 0.00% | 0.00% | 0.00% | 0.00% | 0.00% | 0.00% | 0.00% | 0.00% | 0.00% | 0.00% |
| #4 | HiTOM | 47.03% | 0.00% | 0.00% | 0.00% | 0.00% | 0.00% | 0.00% | 0.00% | 0.00% | 0.00% | 0.00% | 0.00% | 0.00% | 0.00% | 0.00% | 0.00% | 17.24% | 0.00% | 0.00% |
| #5 | HiTOM | 0.00% | 0.00% | 0.00% | 0.00% | 0.00% | 0.00% | 0.00% | 0.00% | 0.00% | 0.00% | 0.00% | 0.00% | 0.00% | 0.00% | 0.00% | 0.00% | 0.00% | 0.00% | 0.00% |
| #6 | HiTOM | 0.00% | 0.00% | 0.00% | 0.00% | 0.00% | 0.00% | 0.00% | 0.00% | 0.00% | 0.00% | 0.00% | 0.00% | 0.00% | 0.00% | 0.00% | 0.00% | 0.00% | 0.00% | 0.00% |
| #7 | HiTOM | 0.00% | 0.00% | 0.00% | 0.00% | 0.00% | 0.00% | 0.00% | 0.00% | 0.00% | 0.00% | 0.00% | 0.00% | 0.00% | 0.00% | 0.00% | 0.00% | 0.00% | 0.00% | 0.00% |
| #8 | HiTOM | 2.97% | 0.00% | 0.00% | 0.00% | 0.00% | 0.00% | 0.00% | 0.00% | 0.00% | 0.00% | 10.11% | 0.00% | 0.00% | 0.00% | 0.00% | 0.00% | 2.23% | 0.00% | 0.00% |
| #9 | HiTOM | 0.00% | 0.00% | 0.00% | 0.00% | 0.00% | 0.00% | 0.00% | 0.00% | 0.00% | 0.00% | 0.00% | 0.00% | 0.00% | 0.00% | 0.00% | 0.00% | 0.00% | 0.00% | 0.00% |
| #10 | HiTOM | 38.90% | 0.00% | 0.00% | 0.00% | 0.00% | 0.00% | 0.00% | 0.00% | 0.00% | 0.00% | 14.30% | 0.00% | 0.00% | 0.00% | 0.00% | 0.00% | 0.00% | 0.00% | 0.00% |
| #11 | HiTOM | 5.36% | 0.00% | 0.00% | 0.00% | 0.00% | 0.00% | 0.00% | 5.14% | 0.00% | 5.14% | 0.00% | 0.00% | 0.00% | 13.72% | 0.00% | 0.00% | 0.00% | 0.00% | 0.00% |
| #12 | HiTOM | 0.00% | 0.00% | 0.00% | 0.00% | 0.00% | 0.00% | 0.00% | 0.00% | 0.00% | 0.00% | 1.41% | 8.20% | 0.00% | 0.00% | 0.00% | 0.00% | 0.00% | 0.00% | 0.00% |
| #13 | HiTOM | 0.00% | 0.00% | 0.00% | 0.00% | 0.00% | 0.00% | 0.00% | 0.00% | 0.00% | 0.00% | 0.00% | 0.00% | 0.00% | 0.00% | 0.00% | 0.00% | 0.00% | 0.00% | 0.00% |
| #14 | HiTOM | 18.36% | 0.00% | 0.00% | 0.00% | 10.37% | 0.00% | 0.00% | 0.00% | 0.00% | 0.00% | 26.50% | 0.00% | 0.00% | 26.50% | 1.76% | 0.00% | 0.00% | 0.00% | 0.00% |
| #15 | HiTOM | 0.00% | 0.00% | 0.00% | 0.00% | 0.00% | 0.00% | 0.00% | 0.00% | 0.00% | 0.00% | 1.57% | 0.00% | 0.00% | 0.00% | 0.00% | 0.00% | 0.00% | 0.00% | 0.00% |
| #16 | HiTOM | 0.00% | 0.00% | 0.00% | 0.00% | 0.00% | 0.00% | 0.00% | 0.00% | 0.00% | 0.00% | 0.00% | 0.00% | 0.00% | 0.00% | 0.00% | 0.00% | 0.00% | 0.00% | 0.00% |
| #17 | HiTOM | 0.00% | 0.00% | 0.00% | 0.00% | 0.00% | 0.00% | 0.00% | 0.00% | 0.00% | 0.00% | 0.00% | 0.00% | 0.00% | 0.00% | 0.00% | 0.00% | 0.00% | 0.00% | 0.00% |
| #18 | HiTOM | 0.00% | 0.00% | 0.00% | 0.00% | 0.00% | 0.00% | 0.00% | 16.20% | 0.00% | 0.00% | 0.00% | 0.00% | 0.00% | 0.00% | 0.00% | 0.00% | 0.00% | 0.00% | 0.00% |
| #19 | HiTOM | 0.00% | 0.00% | 0.00% | 0.00% | 0.00% | 0.00% | 0.00% | 0.00% | 0.00% | 0.00% | 0.00% | 0.00% | 0.00% | 0.00% | 0.00% | 0.00% | 0.00% | 0.00% | 0.00% |
| #20 | HiTOM | 0.00% | 0.00% | 0.00% | 0.00% | 0.00% | 0.00% | 0.00% | 1.27% | 0.00% | 0.00% | 2.11% | 0.00% | 0.00% | 0.00% | 0.00% | 0.00% | 0.00% | 0.00% | 0.00% |
| #21 | HiTOM | 0.00% | 0.00% | 0.00% | 0.00% | 0.00% | 0.00% | 0.00% | 0.00% | 0.00% | 0.00% | 0.00% | 0.00% | 0.00% | 0.00% | 0.00% | 0.00% | 0.00% | 0.00% | 0.00% |
| #22 | HiTOM | 0.00% | 0.00% | 0.00% | 0.00% | 0.00% | 0.00% | 0.00% | 0.00% | 0.00% | 0.00% | 0.00% | 0.00% | 0.00% | 0.00% | 0.00% | 0.00% | 0.00% | 0.00% | 0.00% |
| #23 | HiTOM | 0.00% | 0.00% | 0.00% | 0.00% | 0.00% | 0.00% | 0.00% | 0.00% | 0.00% | 0.00% | 0.00% | 0.00% | 0.00% | 0.00% | 0.00% | 0.00% | 2.22% | 0.00% | 0.00% |
| #24 | HiTOM | 0.00% | 0.00% | 0.00% | 0.00% | 0.00% | 0.00% | 0.00% | 0.00% | 0.00% | 0.00% | 0.00% | 0.00% | 0.00% | 0.00% | 0.00% | 0.00% | 0.00% | 0.00% | 0.00% |
| #25 | HiTOM | 0.00% | 0.00% | 0.00% | 0.00% | 0.00% | 0.00% | 0.00% | 0.00% | 0.00% | 0.00% | 0.00% | 0.00% | 0.00% | 0.00% | 0.00% | 0.00% | 0.00% | 0.00% | 0.00% |
| #26 | HiTOM | 14.09% | 15.97% | 0.00% | 0.00% | 0.00% | 0.00% | 0.00% | 0.00% | 0.00% | 0.00% | 15.97% | 0.00% | 0.00% | 23.48% | 0.00% | 0.00% | 0.00% | 0.00% | 0.00% |
| #27 | HiTOM | 1.22% | 0.00% | 3.90% | 0.00% | 0.00% | 0.00% | 0.00% | 0.00% | 0.00% | 0.00% | 12.02% | 0.00% | 0.00% | 0.00% | 0.00% | 0.00% | 0.00% | 0.00% | 0.00% |
| #28 | HiTOM | 0.00% | 0.00% | 0.00% | 0.00% | 0.00% | 0.00% | 0.00% | 0.00% | 0.00% | 0.00% | 0.00% | 0.00% | 0.00% | 0.00% | 0.00% | 0.00% | 0.00% | 0.00% | 0.00% |
| #29 | HiTOM | 0.00% | 0.00% | 0.00% | 0.00% | 0.00% | 0.00% | 0.00% | 0.00% | 0.00% | 0.00% | 0.00% | 0.00% | 0.00% | 0.00% | 0.00% | 0.00% | 0.00% | 0.00% | 0.00% |
| #30 | HiTOM | 35.78% | 0.00% | 0.00% | 0.00% | 0.00% | 0.00% | 0.00% | 18.85% | 0.00% | 0.00% | 5.06% | 0.00% | 0.00% | 0.00% | 0.00% | 0.00% | 1.59% | 0.00% | 0.00% |
| #31 | HiTOM | 0.00% | 0.00% | 0.00% | 0.00% | 0.00% | 0.00% | 0.00% | 0.00% | 0.00% | 0.00% | 0.00% | 0.00% | 0.00% | 0.00% | 0.00% | 0.00% | 0.00% | 0.00% | 0.00% |
| #32 | HiTOM | 0.00% | 0.00% | 0.00% | 0.00% | 0.00% | 0.00% | 0.00% | 0.00% | 0.00% | 0.00% | 0.00% | 0.00% | 0.00% | 0.00% | 0.00% | 0.00% | 0.00% | 0.00% | 0.00% |

**Supplementary table 3.** Continued.

| **CE_1029_TDG3** | | | **EPSPS-g1** | | |
| --- | --- | --- | --- | --- | --- |
| Total  T0 plants | Genotyping method | InDel | T5 | | |
|  |  |  | T-to-G | T-to-C | T-to-A |
| 20 | Reads%>10% | 0 | 1 | 0 | 0 |
| #1 | HiTOM | 0.00% | 0.00% | 0.00% | 0.00% |
| #2 | HiTOM | 0.00% | 0.00% | 0.00% | 0.00% |
| #3 | HiTOM | 2.01% | 8.13% | 0.00% | 0.00% |
| #4 | HiTOM | 0.00% | 0.00% | 0.00% | 0.00% |
| #5 | HiTOM | 0.00% | 0.00% | 0.00% | 0.00% |
| #6 | HiTOM | 0.00% | 3.54% | 0.00% | 0.00% |
| #7 | HiTOM | 0.00% | 0.00% | 0.00% | 0.00% |
| #8 | HiTOM | 0.00% | 0.00% | 0.00% | 0.00% |
| #9 | HiTOM | 0.00% | 1.91% | 0.00% | 0.00% |
| #10 | HiTOM | 0.00% | 0.00% | 0.00% | 0.00% |
| #11 | HiTOM | 0.00% | 0.00% | 0.00% | 0.00% |
| #12 | HiTOM | 0.00% | 0.00% | 0.00% | 0.00% |
| #13 | HiTOM | 0.00% | 0.00% | 0.00% | 0.00% |
| #14 | HiTOM | 0.00% | 0.00% | 0.00% | 0.00% |
| #15 | HiTOM | 0.00% | 13.49% | 0.00% | 0.00% |
| #16 | HiTOM | 0.00% | 0.00% | 0.00% | 0.00% |
| #17 | HiTOM | 0.00% | 0.00% | 0.00% | 0.00% |
| #18 | HiTOM | 5.97% | 0.00% | 0.00% | 0.00% |
| #19 | HiTOM | 0.00% | 0.00% | 0.00% | 0.00% |
| #20 | HiTOM | 0.00% | 0.00% | 0.00% | 0.00% |

**Supplementary table 3.** Continued.

| **CE_1029_TDG3** | | | **TB1-g1** | | |
| --- | --- | --- | --- | --- | --- |
| Total  T0 plants | Genotyping method | InDel | T3 | | |
|  |  |  | T-to-G | T-to-C | T-to-A |
| 24 | Reads%>10% | 0 | 2 | 0 | 0 |
| #1 | HiTOM | 0.00% | 0.00% | 0.00% | 0.00% |
| #2 | HiTOM | 0.00% | 0.00% | 0.00% | 0.00% |
| #3 | HiTOM | 0.00% | 0.00% | 0.00% | 0.00% |
| #4 | HiTOM | 0.00% | 0.00% | 0.00% | 0.00% |
| #5 | HiTOM | 0.00% | 0.00% | 0.00% | 0.00% |
| #6 | HiTOM | 0.00% | 0.00% | 0.00% | 0.00% |
| #7 | HiTOM | 3.01% | 15.06% | 0.00% | 0.00% |
| #8 | HiTOM | 0.00% | 0.00% | 0.00% | 0.00% |
| #9 | HiTOM | 0.00% | 0.00% | 0.00% | 0.00% |
| #10 | HiTOM | 0.00% | 0.00% | 0.00% | 0.00% |
| #11 | HiTOM | 0.00% | 0.00% | 0.00% | 0.00% |
| #12 | HiTOM | 0.00% | 0.00% | 0.00% | 0.00% |
| #13 | HiTOM | 0.00% | 3.59% | 0.00% | 0.00% |
| #14 | HiTOM | 5.33% | 30.49% | 0.00% | 0.00% |
| #15 | HiTOM | 0.00% | 0.00% | 0.00% | 0.00% |
| #16 | HiTOM | 0.00% | 1.85% | 0.00% | 0.00% |
| #17 | HiTOM | 0.00% | 0.00% | 0.00% | 0.00% |
| #18 | HiTOM | 0.00% | 0.00% | 0.00% | 0.00% |
| #19 | HiTOM | 0.00% | 0.00% | 0.00% | 0.00% |
| #20 | HiTOM | 0.00% | 0.00% | 0.00% | 0.00% |
| #21 | HiTOM | 0.00% | 0.00% | 0.00% | 0.00% |
| #22 | HiTOM | 0.00% | 0.00% | 0.00% | 0.00% |
| #23 | HiTOM | 0.00% | 0.00% | 0.00% | 0.00% |
| #24 | HiTOM | 0.00% | 0.00% | 0.00% | 0.00% |

**Supplementary table 3.** Continued.

| **CE_1046_TDG3** | | | **SLR1-g1** | | |
| --- | --- | --- | --- | --- | --- |
| Total  T0 plants | Genotyping method | InDel | T5 | | |
|  |  |  | T-to-G | T-to-C | T-to-A |
| 31 | Reads%>10% | 8 | 1 | 0 | 0 |
| #1 | HiTOM | 0.00% | 0.00% | 0.00% | 0.00% |
| #2 | HiTOM | 0.00% | 0.00% | 0.00% | 0.00% |
| #3 | HiTOM | 55.31% | 0.00% | 3.89% | 0.00% |
| #4 | HiTOM | 0.00% | 0.00% | 0.00% | 0.00% |
| #5 | HiTOM | 41.49% | 0.00% | 6.08% | 0.00% |
| #6 | HiTOM | 22.46% | 0.00% | 0.00% | 0.00% |
| #7 | HiTOM | 2.25% | 5.75% | 0.00% | 0.00% |
| #8 | HiTOM | 0.00% | 0.00% | 0.00% | 0.00% |
| #9 | HiTOM | 0.00% | 0.00% | 0.00% | 0.00% |
| #10 | HiTOM | 8.87% | 0.00% | 0.00% | 0.00% |
| #11 | HiTOM | 0.00% | 0.00% | 0.00% | 0.00% |
| #12 | HiTOM | 0.00% | 0.00% | 0.00% | 0.00% |
| #13 | HiTOM | 23.18% | 9.27% | 0.00% | 0.00% |
| #14 | HiTOM | 0.00% | 0.00% | 0.00% | 0.00% |
| #15 | HiTOM | 5.45% | 25.81% | 0.00% | 5.35% |
| #16 | HiTOM | 49.33% | 3.35% | 0.00% | 0.00% |
| #17 | HiTOM | 45.07% | 4.91% | 0.00% | 0.00% |
| #18 | HiTOM | 9.40% | 0.00% | 0.00% | 0.00% |
| #19 | HiTOM | 0.00% | 0.00% | 0.00% | 0.00% |
| #20 | HiTOM | 0.00% | 0.00% | 0.00% | 0.00% |
| #21 | HiTOM | 0.00% | 0.00% | 0.00% | 0.00% |
| #22 | HiTOM | 3.31% | 0.00% | 0.00% | 0.00% |
| #23 | HiTOM | 11.95% | 8.19% | 0.00% | 2.74% |
| #24 | HiTOM | 0.00% | 0.00% | 0.00% | 0.00% |
| #25 | HiTOM | 0.00% | 0.00% | 0.00% | 0.00% |
| #26 | HiTOM | 5.19% | 0.00% | 0.00% | 0.00% |
| #27 | HiTOM | 0.00% | 0.00% | 0.00% | 0.00% |
| #28 | HiTOM | 0.00% | 0.00% | 0.00% | 0.00% |
| #29 | HiTOM | 10.37% | 0.00% | 0.00% | 0.00% |
| #30 | HiTOM | 0.00% | 0.00% | 0.00% | 0.00% |
| #31 | HiTOM | 0.00% | 0.00% | 0.00% | 0.00% |

**Supplementary table 3.** Continued.

| **CE_1046_TDG3** | | | **SLR1-g2** | | |
| --- | --- | --- | --- | --- | --- |
| Total  T0 plants | Genotyping method | InDel | T5 | | |
|  |  |  | T-to-G | T-to-C | T-to-A |
| 23 | Reads%>10% | 9 | 3 | 0 | 0 |
| #1 | HiTOM | 0.00% | 0.00% | 0.00% | 0.00% |
| #2 | HiTOM | 0.00% | 0.00% | 0.00% | 0.00% |
| #3 | HiTOM | 14.31% | 9.34% | 0.00% | 2.34% |
| #4 | HiTOM | 25.08% | 25.04% | 0.00% | 0.00% |
| #5 | HiTOM | 27.76% | 35.89% | 0.00% | 0.00% |
| #6 | HiTOM | 0.00% | 0.00% | 0.00% | 0.00% |
| #7 | HiTOM | 8.21% | 0.00% | 0.00% | 0.00% |
| #8 | HiTOM | 0.00% | 0.00% | 0.00% | 0.00% |
| #9 | HiTOM | 0.00% | 0.00% | 0.00% | 0.00% |
| #10 | HiTOM | 42.30% | 5.77% | 0.00% | 0.00% |
| #11 | HiTOM | 0.00% | 0.00% | 0.00% | 0.00% |
| #12 | HiTOM | 2.09% | 0.00% | 0.00% | 0.00% |
| #13 | HiTOM | 48.08% | 3.89% | 0.00% | 0.00% |
| #14 | HiTOM | 11.50% | 0.00% | 0.00% | 0.00% |
| #15 | HiTOM | 0.00% | 0.00% | 0.00% | 0.00% |
| #16 | HiTOM | 0.00% | 0.00% | 0.00% | 0.00% |
| #17 | HiTOM | 0.00% | 6.67% | 0.00% | 0.00% |
| #18 | HiTOM | 19.57% | 29.75% | 0.00% | 0.00% |
| #19 | HiTOM | 0.00% | 0.00% | 0.00% | 0.00% |
| #20 | HiTOM | 0.00% | 0.00% | 0.00% | 0.00% |
| #21 | HiTOM | 45.97% | 2.91% | 0.00% | 0.00% |
| #22 | HiTOM | 3.35% | 0.00% | 0.00% | 0.00% |
| #23 | HiTOM | 39.46% | 0.00% | 0.00% | 0.00% |

**Supplementary table 3.** Continued.

| **CE_1046_TDG3** | | | **SLR1-g3** | | | | | | | | |
| --- | --- | --- | --- | --- | --- | --- | --- | --- | --- | --- | --- |
| Total  T0 plants | Genotyping method | InDel | T6 | | | T9 | | | T11 | | |
|  |  |  | T-to-G | T-to-C | T-to-A | T-to-G | T-to-C | T-to-A | T-to-G | T-to-C | T-to-A |
| 34 | Reads%>10% | 13 | 10 | 0 | 2 | 2 | 0 | 0 | 1 | 0 | 0 |
| #1 | HiTOM | 14.77% | 47.68% | 1.73% | 2.43% | 0.00% | 0.00% | 0.00% | 0.00% | 0.00% | 0.00% |
| #2 | HiTOM | 41.00% | 21.60% | 0.00% | 17.04% | 0.00% | 0.00% | 0.00% | 0.00% | 0.00% | 0.00% |
| #3 | HiTOM | 54.66% | 18.76% | 0.00% | 3.26% | 0.00% | 0.00% | 0.00% | 0.00% | 0.00% | 0.00% |
| #4 | HiTOM | 24.54% | 18.06% | 0.00% | 2.95% | 0.00% | 0.00% | 0.00% | 2.07% | 0.00% | 0.00% |
| #5 | HiTOM | 51.95% | 0.00% | 0.00% | 0.00% | 0.00% | 0.00% | 0.00% | 0.00% | 0.00% | 0.00% |
| #6 | HiTOM | 14.10% | 16.59% | 2.18% | 0.00% | 0.00% | 0.00% | 0.00% | 3.08% | 0.00% | 0.00% |
| #7 | HiTOM | 0.00% |  | 0.00% | 0.00% | 0.00% | 0.00% | 0.00% | 0.00% | 0.00% | 0.00% |
| #8 | HiTOM | 0.00% | 2.80% | 0.00% | 0.00% | 0.00% | 0.00% | 0.00% | 0.00% | 0.00% | 0.00% |
| #9 | HiTOM | 17.84% | 59.55% | 0.00% | 1.40% | 0.00% | 0.00% | 0.00% | 3.14% | 0.00% | 0.00% |
| #10 | HiTOM | 0.00% | 0.00% | 0.00% | 0.00% | 0.00% | 0.00% | 0.00% | 0.00% | 0.00% | 0.00% |
| #11 | HiTOM | 0.00% | 0.00% | 0.00% | 0.00% | 0.00% | 0.00% | 0.00% | 0.00% | 0.00% | 0.00% |
| #12 | HiTOM | 0.00% | 0.00% | 1.04% | 0.00% | 0.00% | 0.00% | 0.00% | 0.00% | 0.00% | 0.00% |
| #13 | HiTOM | 0.00% | 0.00% | 1.94% | 0.00% | 0.00% | 0.00% | 0.00% | 0.00% | 0.00% | 0.00% |
| #14 | HiTOM | 0.00% | 0.00% | 0.00% | 0.00% | 0.00% | 0.00% | 0.00% | 0.00% | 0.00% | 0.00% |
| #15 | HiTOM | 0.00% | 0.00% | 0.00% | 0.00% | 0.00% | 0.00% | 0.00% | 0.00% | 0.00% | 0.00% |
| #16 | HiTOM | 27.32% | 15.01% | 0.00% | 0.00% | 0.00% | 0.00% | 0.00% | 0.00% | 0.00% | 0.00% |
| #17 | HiTOM | 0.00% | 0.00% | 0.00% | 0.00% | 0.00% | 0.00% | 0.00% | 0.00% | 0.00% | 0.00% |
| #18 | HiTOM | 46.25% | 1.14% | 0.00% | 0.00% | 40.92% | 0.00% | 0.00% | 0.00% | 0.00% | 0.00% |
| #19 | HiTOM | 29.28% | 30.06% | 0.00% | 32.45% | 1.61% | 0.00% | 4.84% | 0.00% | 0.00% | 0.00% |
| #20 | HiTOM | 9.63% | 10.80% | 0.00% | 0.00% | 0.00% | 0.00% | 0.00% | 0.00% | 0.00% | 0.00% |
| #21 | HiTOM | 19.27% | 4.09% | 0.00% | 0.00% | 0.00% | 0.00% | 0.00% | 0.00% | 0.00% | 0.00% |
| #22 | HiTOM | 0.00% | 0.00% | 0.00% | 0.00% | 0.00% | 0.00% | 0.00% | 0.00% | 0.00% | 0.00% |
| #23 | HiTOM | 9.87% | 0.00% | 0.00% | 0.00% | 0.00% | 1.74% | 0.00% | 8.27% | 0.00% | 0.00% |
| #24 | HiTOM | 3.41% | 6.71% | 0.00% | 0.00% | 0.00% | 0.00% | 0.00% | 0.00% | 0.00% | 0.00% |
| #25 | HiTOM | 0.00% | 0.00% | 0.00% | 0.00% | 0.00% | 0.00% | 0.00% | 0.00% | 0.00% | 0.00% |
| #26 | HiTOM | 1.34% | 0.00% | 0.00% | 0.00% | 1.15% | 0.00% | 0.00% | 0.00% | 0.00% | 0.00% |
| #27 | HiTOM | 0.00% | 0.00% | 0.00% | 0.00% | 14.23% | 0.00% | 0.00% | 0.00% | 0.00% | 0.00% |
| #28 | HiTOM | 2.49% | 12.05% | 1.61% | 1.22% | 0.00% | 0.00% | 0.00% | 0.00% | 0.00% | 0.00% |
| #29 | HiTOM | 0.00% | 2.19% | 0.00% | 0.00% | 0.00% | 0.00% | 0.00% | 0.00% | 0.00% | 0.00% |
| #30 | HiTOM | 0.00% | 1.41% | 0.00% | 0.00% | 0.00% | 1.06% | 0.00% | 0.00% | 0.00% | 0.00% |
| #31 | HiTOM | 1.01% | 7.88% | 0.00% | 0.00% | 0.00% | 0.00% | 0.00% | 0.00% | 0.00% | 0.00% |
| #32 | HiTOM | 41.73% | 3.54% | 0.00% | 0.00% | 0.00% | 0.00% | 0.00% | 0.00% | 0.00% | 0.00% |
| #33 | HiTOM | 9.20% | 0.00% | 0.00% | 0.00% | 0.00% | 0.00% | 0.00% | 29.56% | 0.00% | 0.00% |
| #34 | HiTOM | 64.39% | 8.69% | 0.00% | 1.57% | 0.00% | 0.00% | 0.00% | 0.00% | 0.00% | 0.00% |

**Supplementary table 3.** Continued.

| **CE_1046_TDG3** | | | **ALS1-g1** | | |
| --- | --- | --- | --- | --- | --- |
| Total  T0 plants | Genotyping method | InDel | T-1 | | |
|  |  |  | T-to-G | T-to-C | T-to-A |
| 11 | Reads%>10% | 2 | 1 | 0 | 0 |
| #1 | HiTOM | 0.00% | 0.00% | 0.00% | 0.00% |
| #2 | HiTOM | 35.09% | 3.17% | 0.00% | 0.00% |
| #3 | HiTOM | 0.00% | 0.00% | 0.00% | 0.00% |
| #4 | HiTOM | 0.00% | 5.35% | 0.00% | 0.00% |
| #5 | HiTOM | 0.00% | 0.00% | 0.00% | 0.00% |
| #6 | HiTOM | 17.94% | 18.49% | 0.00% | 0.00% |
| #7 | HiTOM | 2.59% | 0.00% | 0.00% | 0.00% |
| #8 | HiTOM | 0.00% | 0.00% | 0.00% | 0.00% |
| #9 | HiTOM | 0.00% | 3.04% | 0.00% | 0.00% |
| #10 | HiTOM | 9.11% | 0.00% | 0.00% | 0.00% |
| #11 | HiTOM | 0.00% | 0.00% | 0.00% | 0.00% |

**Supplementary table 3.** Continued.

| **CE_1046_TDG3** | | | **EPSPS-g1** | | |
| --- | --- | --- | --- | --- | --- |
| Total  T0 plants | Genotyping method | InDel | T5 | | |
|  |  |  | T-to-G | T-to-C | T-to-A |
| 35 | Reads%>10% | 12 | 4 | 0 | 0 |
| #1 | HiTOM | 44.79% | 1.47% | 0.00% | 0.00% |
| #2 | HiTOM | 31.71% | 10.76% | 0.00% | 0.00% |
| #3 | HiTOM | 13.82% | 2.97% | 0.00% | 0.00% |
| #4 | HiTOM | 20.06% | 47.32% | 0.00% | 0.00% |
| #5 | HiTOM | 51.98% | 0.00% | 0.00% | 0.00% |
| #6 | HiTOM | 0.00% | 0.00% | 0.00% | 0.00% |
| #7 | HiTOM | 7.10% | 1.49% | 0.00% | 0.00% |
| #8 | HiTOM | 0.00% | 0.00% | 2.35% | 0.00% |
| #9 | HiTOM | 7.25% | 9.92% | 0.00% | 0.00% |
| #10 | HiTOM | 0.00% | 1.97% | 0.00% | 0.00% |
| #11 | HiTOM | 0.00% | 0.00% | 0.00% | 0.00% |
| #12 | HiTOM | 0.00% | 0.00% | 0.00% | 0.00% |
| #13 | HiTOM | 0.00% | 0.00% | 0.00% | 0.00% |
| #14 | HiTOM | 51.91% | 0.00% | 0.00% | 0.00% |
| #15 | HiTOM | 0.00% | 0.00% | 0.00% | 0.00% |
| #16 | HiTOM | 50.59% | 0.00% | 0.00% | 0.00% |
| #17 | HiTOM | 28.10% | 1.83% | 0.00% | 0.00% |
| #18 | HiTOM | 0.00% | 47.06% | 0.00% | 0.00% |
| #19 | HiTOM | 0.00% | 2.05% | 0.00% | 0.00% |
| #20 | HiTOM | 0.00% | 0.00% | 0.00% | 0.00% |
| #21 | HiTOM | 15.30% | 2.00% | 0.00% | 0.00% |
| #22 | HiTOM | 45.55% | 2.11% | 0.00% | 0.00% |
| #23 | HiTOM | 0.00% | 0.00% | 0.00% | 0.00% |
| #24 | HiTOM | 0.00% | 0.00% | 0.00% | 0.00% |
| #25 | HiTOM | 11.40% | 1.66% | 0.00% | 0.00% |
| #26 | HiTOM | 4.72% | 9.10% | 0.00% | 0.00% |
| #27 | HiTOM | 0.00% | 2.09% | 0.00% | 0.00% |
| #28 | HiTOM | 0.00% | 1.50% | 0.00% | 0.00% |
| #29 | HiTOM | 2.44% | 0.00% | 0.00% | 0.00% |
| #30 | HiTOM | 0.00% | 72.58% | 0.00% | 0.00% |
| #31 | HiTOM | 0.00% | 0.00% | 0.00% | 0.00% |
| #32 | HiTOM | 10.94% | 3.43% | 0.00% | 0.00% |
| #33 | HiTOM | 0.00% | 0.00% | 0.00% | 0.00% |
| #34 | HiTOM | 0.00% | 0.00% | 0.00% | 0.00% |
| #35 | HiTOM | 0.00% | 0.00% | 0.00% | 0.00% |

**Supplementary table 3.** Continued.

| **CE_1046_TDG3** | | | **TB1-g1** | | | | | | | | |
| --- | --- | --- | --- | --- | --- | --- | --- | --- | --- | --- | --- |
| Total  T0 plants | Genotyping method | InDel | T3 | | | T4 | | | T9 | | |
|  |  |  | T-to-G | T-to-C | T-to-A | T-to-G | T-to-C | T-to-A | T-to-G | T-to-C | T-to-A |
| 31 | Reads%>10% | 4 | 3 | 0 | 0 | 0 | 0 | 0 | 2 | 0 | 0 |
| #1 | HiTOM | 0.00% | 0.00% | 0.00% | 0.00% | 0.00% | 0.00% | 0.00% | 0.00% | 0.00% | 0.00% |
| #2 | HiTOM | 33.35% | 0.00% | 0.00% | 0.00% | 0.00% | 0.00% | 0.00% | 0.00% | 0.00% | 0.00% |
| #3 | HiTOM | 0.00% | 11.69% | 0.00% | 0.00% | 3.65% | 0.00% | 0.00% | 5.87% | 0.00% | 0.00% |
| #4 | HiTOM | 8.48% | 3.35% | 0.00% | 0.00% | 0.00% | 0.00% | 0.00% | 0.00% | 0.00% | 0.00% |
| #5 | HiTOM | 45.31% | 2.14% | 0.00% | 0.00% | 0.00% | 0.00% | 0.00% | 0.00% | 0.00% | 0.00% |
| #6 | HiTOM | 0.00% | 24.06% | 0.00% | 0.00% | 0.00% | 0.00% | 0.00% | 0.00% | 0.00% | 0.00% |
| #7 | HiTOM | 0.00% | 0.00% | 0.00% | 0.00% | 0.00% | 0.00% | 0.00% | 0.00% | 0.00% | 0.00% |
| #8 | HiTOM | 3.19% | 0.00% | 0.00% | 6.38% | 0.00% | 0.00% | 0.00% | 1.58% | 0.00% | 0.00% |
| #9 | HiTOM | 0.00% | 0.00% | 0.00% | 0.00% | 0.00% | 0.00% | 0.00% | 0.00% | 0.00% | 0.00% |
| #10 | HiTOM | 0.00% | 0.00% | 0.00% | 0.00% | 0.00% | 0.00% | 0.00% | 0.00% | 0.00% | 0.00% |
| #11 | HiTOM | 0.00% | 1.59% | 0.00% | 0.00% | 0.00% | 0.00% | 0.00% | 0.00% | 0.00% | 0.00% |
| #12 | HiTOM | 0.00% | 0.00% | 0.00% | 0.00% | 0.00% | 0.00% | 0.00% | 0.00% | 0.00% | 0.00% |
| #13 | HiTOM | 0.00% | 0.00% | 0.00% | 0.00% | 0.00% | 0.00% | 0.00% | 0.00% | 0.00% | 0.00% |
| #14 | HiTOM | 0.00% | 0.00% | 0.00% | 0.00% | 0.00% | 0.00% | 0.00% | 0.00% | 0.00% | 0.00% |
| #15 | HiTOM | 1.89% | 1.42% | 0.00% | 0.00% | 1.42% | 0.00% | 0.00% | 15.96% | 0.00% | 0.00% |
| #16 | HiTOM | 0.00% | 0.00% | 0.00% | 0.00% | 0.00% | 0.00% | 0.00% | 0.00% | 0.00% | 0.00% |
| #17 | HiTOM | 0.00% | 0.00% | 0.00% | 0.00% | 0.00% | 0.00% | 0.00% | 0.00% | 0.00% | 0.00% |
| #18 | HiTOM | 0.00% | 0.00% | 0.00% | 0.00% | 0.00% | 0.00% | 0.00% | 0.00% | 0.00% | 0.00% |
| #19 | HiTOM | 0.00% | 0.00% | 0.00% | 0.00% | 0.00% | 0.00% | 0.00% | 0.00% | 0.00% | 0.00% |
| #20 | HiTOM | 0.00% | 0.00% | 0.00% | 0.00% | 0.00% | 0.00% | 0.00% | 0.00% | 0.00% | 0.00% |
| #21 | HiTOM | 14.27% | 0.00% | 0.00% | 0.00% | 0.00% | 0.00% | 0.00% | 9.31% | 0.00% | 0.00% |
| #22 | HiTOM | 0.00% | 0.00% | 0.00% | 0.00% | 0.00% | 0.00% | 0.00% | 0.00% | 0.00% | 0.00% |
| #23 | HiTOM | 0.00% | 0.00% | 0.00% | 0.00% | 0.00% | 0.00% | 0.00% | 0.00% | 0.00% | 0.00% |
| #24 | HiTOM | 2.24% | 5.68% | 0.00% | 0.00% | 0.00% | 0.00% | 0.00% | 45.08% | 0.00% | 0.00% |
| #25 | HiTOM | 5.24% | 3.10% | 0.00% | 0.00% | 0.00% | 0.00% | 0.00% | 0.00% | 0.00% | 0.00% |
| #26 | HiTOM | 0.00% | 0.00% | 0.00% | 0.00% | 0.00% | 0.00% | 0.00% | 0.00% | 0.00% | 0.00% |
| #27 | HiTOM | 0.00% | 0.00% | 0.00% | 0.00% | 0.00% | 0.00% | 0.00% | 0.00% | 0.00% | 0.00% |
| #28 | HiTOM | 10.53% | 13.84% | 0.00% | 3.97% | 1.56% | 0.00% | 0.00% | 0.00% | 0.00% | 0.00% |
| #29 | HiTOM | 0.00% | 0.00% | 0.00% | 0.00% | 0.00% | 0.00% | 0.00% | 0.00% | 0.00% | 0.00% |
| #30 | HiTOM | 0.00% | 0.00% | 0.00% | 0.00% | 0.00% | 0.00% | 0.00% | 0.00% | 0.00% | 0.00% |
| #31 | HiTOM | 0.00% | 0.00% | 0.00% | 0.00% | 0.00% | 0.00% | 0.00% | 0.00% | 0.00% | 0.00% |

**Supplementary table 3.** Continued.

| **CE_1249_TDG3** | | | **SLR1-g2** | | |
| --- | --- | --- | --- | --- | --- |
| Total  T0 plants | Genotyping method | InDel | T8 | | |
|  |  |  | T-to-G | T-to-C | T-to-A |
| 15 | Reads%>10% | 1 | 0 | 0 | 0 |
| #1 | HiTOM | 0.00% | 0.00% | 0.00% | 0.00% |
| #2 | HiTOM | 0.00% | 0.00% | 1.91% | 0.00% |
| #3 | HiTOM | 0.00% | 0.00% | 0.00% | 0.00% |
| #4 | HiTOM | 0.00% | 0.00% | 0.00% | 0.00% |
| #5 | HiTOM | 0.00% | 0.00% | 0.00% | 0.00% |
| #6 | HiTOM | 2.49% | 3.85% | 0.00% | 0.00% |
| #7 | HiTOM | 0.00% | 0.00% | 0.00% | 0.00% |
| #8 | HiTOM | 0.00% | 0.00% | 0.00% | 0.00% |
| #9 | HiTOM | 31.31% | 8.14% | 0.00% | 0.00% |
| #10 | HiTOM | 5.57% | 0.00% | 0.00% | 0.00% |
| #11 | HiTOM | 0.00% | 0.00% | 0.00% | 0.00% |
| #12 | HiTOM | 0.00% | 0.00% | 0.00% | 0.00% |
| #13 | HiTOM | 0.00% | 0.00% | 0.00% | 0.00% |
| #14 | HiTOM | 0.00% | 0.00% | 0.00% | 0.00% |
| #15 | HiTOM | 0.00% | 0.00% | 0.00% | 0.00% |

**Supplementary table 3.** Continued.

| **CE_1249_TDG3** | | | **SLR1-g3** | | |
| --- | --- | --- | --- | --- | --- |
| Total  T0 plants | Genotyping method | InDel | T11 | | |
|  |  |  | T-to-G | T-to-C | T-to-A |
| 23 | Reads%>10% | 2 | 2 | 0 | 0 |
| #1 | HiTOM | 0.00% | 0.00% | 0.00% | 0.00% |
| #2 | HiTOM | 0.00% | 0.00% | 0.00% | 0.00% |
| #3 | HiTOM | 0.00% | 0.00% | 0.00% | 0.00% |
| #4 | HiTOM | 0.00% | 0.00% | 0.00% | 0.00% |
| #5 | HiTOM | 8.85% | 2.26% | 0.00% | 0.00% |
| #6 | HiTOM | 27.39% | 15.39% | 0.00% | 0.00% |
| #7 | HiTOM | 1.89% | 0.00% | 0.00% | 0.00% |
| #8 | HiTOM | 0.00% | 0.00% | 0.00% | 0.00% |
| #9 | HiTOM | 0.00% | 0.00% | 0.00% | 0.00% |
| #10 | HiTOM | 0.00% | 0.00% | 0.00% | 0.00% |
| #11 | HiTOM | 0.00% | 0.00% | 0.00% | 0.00% |
| #12 | HiTOM | 0.00% | 1.85% | 0.00% | 0.00% |
| #13 | HiTOM | 0.00% | 0.00% | 0.00% | 0.00% |
| #14 | HiTOM | 0.00% | 0.00% | 0.00% | 0.00% |
| #15 | HiTOM | 0.00% | 0.00% | 0.00% | 0.00% |
| #16 | HiTOM | 0.00% | 0.00% | 0.00% | 0.00% |
| #17 | HiTOM | 33.04% | 18.27% | 0.00% | 0.00% |
| #18 | HiTOM | 3.33% | 0.00% | 0.00% | 0.00% |
| #19 | HiTOM | 6.36% | 9.66% | 0.00% | 0.00% |
| #20 | HiTOM | 0.00% | 0.00% | 0.00% | 0.00% |
| #21 | HiTOM | 0.00% | 0.00% | 0.00% | 0.00% |
| #22 | HiTOM | 0.00% | 0.00% | 0.00% | 0.00% |
| #23 | HiTOM | 0.00% | 0.00% | 0.00% | 0.00% |

**Supplementary table 3.** Continued.

| **CE_1249_TDG3** | | | **ALS1-g1** | | | | | |
| --- | --- | --- | --- | --- | --- | --- | --- | --- |
| Total  T0 plants | Genotyping method | InDel | T-5 | | | T11 | | |
|  |  |  | T-to-G | T-to-C | T-to-A | T-to-G | T-to-C | T-to-A |
| 17 | Reads%>10% | 3 | 1 | 0 | 0 | 1 | 0 | 0 |
| #1 | HiTOM | 0.00% | 1.09% | 0.00% | 0.00% | 0.00% | 0.00% | 0.00% |
| #2 | HiTOM | 39.08% | 9.48% | 0.00% | 0.00% | 0.00% | 0.00% | 0.00% |
| #3 | HiTOM | 5.19% | 0.00% | 0.00% | 0.00% | 0.00% | 0.00% | 0.00% |
| #4 | HiTOM | 0.00% | 0.00% | 0.00% | 0.00% | 2.19% | 0.00% | 0.00% |
| #5 | HiTOM | 8.18% | 12.14% | 0.00% | 0.00% | 35.03% | 0.00% | 0.00% |
| #6 | HiTOM | 0.00% | 0.00% | 0.00% | 0.00% | 0.00% | 0.00% | 0.00% |
| #7 | HiTOM | 0.00% | 0.00% | 0.00% | 0.00% | 8.34% | 0.00% | 0.00% |
| #8 | HiTOM | 0.00% | 0.00% | 0.00% | 0.00% | 0.00% | 0.00% | 0.00% |
| #9 | HiTOM | 3.15% | 0.00% | 0.00% | 0.00% | 0.00% | 0.00% | 0.00% |
| #10 | HiTOM | 35.19% | 5.47% | 0.00% | 0.00% | 0.00% | 0.00% | 0.00% |
| #11 | HiTOM | 0.00% | 0.00% | 0.00% | 0.00% | 0.00% | 0.00% | 0.00% |
| #12 | HiTOM | 0.00% | 0.00% | 0.00% | 0.00% | 0.00% | 0.00% | 0.00% |
| #13 | HiTOM | 0.00% | 0.00% | 0.00% | 0.00% | 0.00% | 0.00% | 0.00% |
| #14 | HiTOM | 29.39% | 3.65% | 0.00% | 0.00% | 0.00% | 0.00% | 0.00% |
| #15 | HiTOM | 1.96% | 0.00% | 0.00% | 0.00% | 0.00% | 0.00% | 0.00% |
| #16 | HiTOM | 0.00% | 0.00% | 0.00% | 0.00% | 0.00% | 0.00% | 0.00% |
| #17 | HiTOM | 0.00% | 0.00% | 0.00% | 0.00% | 0.00% | 0.00% | 0.00% |

**Supplementary table 3.** Continued.

| **CE_1249_TDG3** | | | **EPSPS-g1** | | |
| --- | --- | --- | --- | --- | --- |
| Total  T0 plants | Genotyping method | InDel | T14 | | |
|  |  |  | T-to-G | T-to-C | T-to-A |
| 23 | Reads%>10% | 1 | 1 | 0 | 0 |
| #1 | HiTOM | 0.00% | 0.00% | 0.00% | 0.00% |
| #2 | HiTOM | 0.00% | 0.00% | 0.00% | 0.00% |
| #3 | HiTOM | 0.00% | 0.00% | 0.00% | 0.00% |
| #4 | HiTOM | 2.88% | 14.19% | 0.00% | 0.00% |
| #5 | HiTOM | 0.00% | 0.00% | 0.00% | 0.00% |
| #6 | HiTOM | 0.00% | 0.00% | 0.00% | 0.00% |
| #7 | HiTOM | 0.00% | 0.00% | 0.00% | 5.49% |
| #8 | HiTOM | 0.00% | 0.00% | 0.00% | 0.00% |
| #9 | HiTOM | 0.00% | 0.00% | 0.00% | 0.00% |
| #10 | HiTOM | 13.35% | 8.59% | 0.00% | 0.00% |
| #11 | HiTOM | 0.00% | 0.00% | 0.00% | 0.00% |
| #12 | HiTOM | 0.00% | 0.00% | 0.00% | 0.00% |
| #13 | HiTOM | 0.00% | 0.00% | 0.00% | 0.00% |
| #14 | HiTOM | 0.00% | 0.00% | 0.00% | 0.00% |
| #15 | HiTOM | 0.00% | 0.00% | 0.00% | 0.00% |
| #16 | HiTOM | 9.83% | 6.69% | 0.00% | 0.00% |
| #17 | HiTOM | 0.00% | 0.00% | 0.00% | 0.00% |
| #18 | HiTOM | 0.00% | 0.00% | 0.00% | 0.00% |
| #19 | HiTOM | 0.00% | 0.00% | 0.00% | 0.00% |
| #20 | HiTOM | 0.00% | 0.00% | 0.00% | 0.00% |
| #21 | HiTOM | 0.00% | 0.00% | 0.00% | 0.00% |
| #22 | HiTOM | 0.00% | 0.00% | 0.00% | 0.00% |
| #23 | HiTOM | 0.00% | 0.00% | 0.00% | 0.00% |

**Supplementary table 3.** Continued.

| **CE_1249_TDG3** | | | **TB1-g1** | | |
| --- | --- | --- | --- | --- | --- |
| Total  T0 plants | Genotyping method | InDel | T9 | | |
|  |  |  | T-to-G | T-to-C | T-to-A |
| 32 | Reads%>10% | 0 | 2 | 0 | 0 |
| #1 | HiTOM | 0.00% | 0.00% | 0.00% | 0.00% |
| #2 | HiTOM | 0.00% | 0.00% | 0.00% | 0.00% |
| #3 | HiTOM | 0.00% | 43.77% | 0.00% | 0.00% |
| #4 | HiTOM | 0.00% | 0.00% | 0.00% | 0.00% |
| #5 | HiTOM | 0.00% | 0.00% | 0.00% | 0.00% |
| #6 | HiTOM | 0.00% | 0.00% | 0.00% | 0.00% |
| #7 | HiTOM | 0.00% | 0.00% | 0.00% | 0.00% |
| #8 | HiTOM | 0.00% | 0.00% | 0.00% | 0.00% |
| #9 | HiTOM | 0.00% | 0.00% | 0.00% | 0.00% |
| #10 | HiTOM | 0.00% | 0.00% | 0.00% | 0.00% |
| #11 | HiTOM | 0.00% | 0.00% | 0.00% | 0.00% |
| #12 | HiTOM | 0.00% | 0.00% | 0.00% | 0.00% |
| #13 | HiTOM | 0.00% | 3.95% | 0.00% | 0.00% |
| #14 | HiTOM | 0.00% | 0.00% | 0.00% | 0.00% |
| #15 | HiTOM | 0.00% | 0.00% | 0.00% | 0.00% |
| #16 | HiTOM | 0.00% | 0.00% | 0.00% | 0.00% |
| #17 | HiTOM | 0.00% | 0.00% | 0.00% | 0.00% |
| #18 | HiTOM | 0.00% | 0.00% | 0.00% | 0.00% |
| #19 | HiTOM | 0.00% | 0.00% | 0.00% | 0.00% |
| #20 | HiTOM | 0.00% | 0.00% | 0.00% | 0.00% |
| #21 | HiTOM | 0.00% | 0.00% | 0.00% | 0.00% |
| #22 | HiTOM | 0.00% | 0.00% | 0.00% | 0.00% |
| #23 | HiTOM | 0.00% | 0.00% | 0.00% | 0.00% |
| #24 | HiTOM | 0.00% | 0.00% | 0.00% | 0.00% |
| #25 | HiTOM | 0.00% | 0.00% | 0.00% | 0.00% |
| #26 | HiTOM | 0.00% | 0.00% | 0.00% | 0.00% |
| #27 | HiTOM | 0.00% | 0.00% | 0.00% | 0.00% |
| #28 | HiTOM | 0.00% | 0.00% | 0.00% | 0.00% |
| #29 | HiTOM | 0.00% | 42.52% | 0.00% | 0.00% |
| #30 | HiTOM | 0.00% | 0.00% | 0.00% | 0.00% |
| #31 | HiTOM | 0.00% | 0.00% | 0.00% | 0.00% |
| #32 | HiTOM | 0.00% | 0.00% | 0.00% | 0.00% |

**Supplementary table 4.** Summary of the off-target sites tested in this study.

Note: ^1^ The tested T_0_ plants including the plants transformed with CE_1029_TDG3, CE_1046_TDG3, and CE_1249_TDG3. ^2^ Mutations were detected by Hi-TOM with the threshold >10%. The NGS raw data was deposited in the National Genomics Data Center (NGDC; https://ngdc.cncb.ac.cn/) under the accession number PRJCA034519.

| **sgRNA** | **Sequence (5'-3')** | **Putative off-target**  **locus** | **T_0_ plants transformed with CE-TDG3^1^** | **Mutated plants^2^** |
| --- | --- | --- | --- | --- |
| **SLR1-g1** | CCCCTCGGACCTCTCCTCCT**GGG** | — | — | — |
| OTSG-1 | CGCCTCGGACACCTCCTCCT**CGG** | chr02:30365512 | 91 | 0 |
| OTSG-2 | CCCCTCCCTCCTCTCCTCCT**GGG** | chr09:13608678 | 91 | 0 |
| OTSG-3 | CTCCTCCATCCTCTCCTCCT**CGG** | chr05:18467268 | 91 | 0 |
| **SLR1-g2** | GTTGTAGTGCACGGTGTCCG**TGG** | — | — | — |
| OTSG-4 | CTTGTAGTGCACGGTGAACA**CGG** | chr06:31201231 | 71 | 0 |
| OTSG-5 | GTGGTGGTACACGGTGTCCT**GGG** | chr11:14203397 | 71 | 0 |
| OTSG-6 | GTCGGAGAGCACGGTGTACG**AGG** | chr01:19969753 | 71 | 0 |
| **SLR1-g3** | AAGCATGCTCTCGACCCAGG**AGG** | — | — | — |
| OTSG-7 | AAGCATGCTCTGGACCCAAA**TGG** | chr06:21554170 | 75 | 0 |
| OTSG-8 | AAGCTTTCTCTCGACACAGG**CAG** | chr01:24508135 | 75 | 0 |
| OTSG-9 | AAGCATTCTCTCGAGCCAAG**GGG** | chr03:10590054 | 75 | 0 |
| **ALS1-g1** | GCGCCCCCACTTGGGATCAT**AGG** | — | — | — |
| OTSG-10 | AGGCCACCACTGGGGATCAT**TGG** | chr09:15887009 | 60 | 0 |
| OTSG-11 | GCGCTCCCTCTTGGAATCTT**GGG** | chr08:15397308 | 60 | 0 |
| OTSG-12 | GAGCTCTCACTGGGGATCAT**TGG** | chr04:31932501 | 60 | 0 |
| **EPSPS-g1** | GCCCTCTCCGAGGTGAGACG**CGG** | — | — | — |
| OTSG-13 | GTCCTCTTCGAGGTGACACG**AAG** | chr06:9124752 | 78 | 0 |
| OTSG-14 | GCTCTCTCCAAGGTGAGTCA**TGG** | chr11:15512681 | 78 | 0 |
| OTSG-15 | GTCCTGTCCGGGGTGAGGCG**GGG** | chr3:4945425 | 78 | 0 |
| **TB1-g1** | ACTTGGAGTTGGAGCCGCAT**GGG** | — | — | — |
| OTSG-16 | AGTTGGAGTTGGAGCTGCTT**CGG** | chr11:27895456 | 87 | 0 |
| OTSG-17 | ACTTGGAGTCGGAGCAGCAG**CGG** | chr7:29404578 | 87 | 0 |
| OTSG-18 | TTTTGGAGTTGGAGCTGCAT**CAG** | chr7:9425195 | 87 | 0 |

**Supplementary table 5.** Heritability analysis on T_1_ progenies.

| **T_0_ Parent lines** | | | **Progeny** | | | |
| --- | --- | --- | --- | --- | --- | --- |
| **Base editor** | **No.** | **Hi-TOM of Base editing^1^** | **Total** | **Base edited T_1_** | | **Transmission rate** |
|  |  |  |  | **Thymine Edited** | **T-DNA free^2^** |  |
| CE1029TDG3 | SLR1-g2#13 | 36.72% | 24 | 9 | 2 | 37.5% |
|  | SLR1-g3#7 | 13.84% | 24 | 0 | 0 | 0% |
|  | ALS1-g1#4 | 17.24% | 24 | 2 | 0 | 8.3% |
|  | TB1-g1#14 | 30.49% | 24 | 10 | 5 | 41.7% |
| CE1046TDG3 | SLR1-g1#15 | 25.81% | 24 | 5 | 0 | 20.8% |
|  | SLR1-g2#5 | 35.89% | 24 | 8 | 3 | 33.3% |
|  | SLR1-g3#1 | 47.68% | 24 | 13 | 7 | 54.2% |
|  | ALS1-g1#6 | 18.49% | 24 | 4 | 0 | 16.7% |
|  | EPSPS-g1#30 | 72.58% | 24 | 20 | 8 | 83.3% |
|  | TB1-g1#24 | 45.08% | 24 | 11 | 4 | 45.8% |
| CE1249TDG3 | SLR1-g3#6 | 15.39% | 24 | 0 | 0 | 0% |
|  | TB1-g1#3 | 43.77% | 24 | 12 | 2 | 50% |

Note: ^1^ Hi-TOM results indicate the ratio of thymine base conversions to total sequencing reads. ^2^ The T-DNA free lines were characterized by PCR with primer pair HYG-F1+HYG-R1.

**Supplementary table 6.** Primers and oligos used in this study.

| **Primer** | **Sequence (5'-3')** | **Function** |
| --- | --- | --- |
| CE1029-F1 | TTGTTTGGTGTTACTTCTGCAGG | Amplifying N-terminal fragment of CE1029 from OsGTBE (Tian et al., 2024) |
| CE1029-R1 | CACTACCGCCTCCTCCGATTTCCTGCTCGCTCTTG |  |
| CE1029-F2 | ACTCGGAGGTGGTGGTTCAGGCAAGGCTACCGCCAAGTAC | Amplifying C-terminal fragment of CE1029 pRABE (Hua et al., 2018) |
| CE1029-R2 | GGAAATTCGAGCTCTATCGATCAATCAG |  |
| CE1046-F1 | TTGTTTGGTGTTACTTCTGCAGG | Amplifying N-terminal fragment of CE1046 from OsGTBE (Tian et al., 2024) |
| CE1046-R1 | CACTACCGCCTCCTCCGAAAAAGTTCATGATGTTGCTGTAGAAG |  |
| CE1046-F2 | CTCGGAGGTGGTGGTTCAATCGAGACAAACGGCGAAACC | Amplifying C-terminal fragment of CE1046 pRABE (Hua et al., 2018) |
| CE1046-R2 | GGAAATTCGAGCTCTATCGATCAATCAG |  |
| CE1249-F1 | TTGTTTGGTGTTACTTCTGCAGG | Amplifying N-terminal fragment of CE1249 from OsGTBE (Tian et al., 2024) |
| CE1249-R1 | CACTACCGCCTCCTCCGGGGGAGCCCTTCAGCTTCTCATAGT |  |
| CE1249-F2 | AACTCGGAGGTGGTGGTTCAGAGGATAATGAGCAGAAACAGCTG | Amplifying C-terminal fragment of CE1249 pRABE (Hua et al., 2018) |
| CE1249-R2 | GGAAATTCGAGCTCTATCGATCAATCAG |  |
| Glycosylase-F | GGAGGAGGCGGTAGTGGGGTG | Amplifying hUNG, hTDG, TDG-EK, and TDG3 |
| Glycosylase-R | TGAACCACCACCTCCGAGTTCC |  |
| G107E-F | TCATGGAGTTCGTGGCAGAGGAAAG | Construction of hTDG and TDG-EK variants |
| G107E-R | CCACGAACTCCATGAGCTTTATGAAG |  |
| Y147A-F | ACCCCGCGCATGGACCTAATC |  |
| Y147A-R | GTCCATGCGCGGGGTCCTGTCCCAA |  |
| R260K-F | ACAGAAAAAAGCACCACGTTCTGCA |  |
| R260K-R | TGGTGCTTTTTTCTGTCGATGGCGGACC |  |
| Hi-SLR-F | GGAGTGAGTACGGTGTGCGGATGACGGGTTCGTGTCGC | Primers for genotyping |
| Hi-SLR-R | GAGTTGGATGCTGGATGGCAGTGGACGAGGTGGAAGC |  |
| Hi-ALS-F | GGAGTGAGTACGGTGTGCCGCCATCAAGAAGATGCT |  |
| Hi-ALS-R | GAGTTGGATGCTGGATGGTGCTTTGCCAACATACAGATTAT |  |
| Hi-EPS-F | GGAGTGAGTACGGTGTGCGAGATCGTGCTCCAGCCCAT |  |
| Hi-EPS-R | GAGTTGGATGCTGGATGGCCACCTCACCTAATAAACCCCCTAA |  |
| Hi-TB-F | GGAGTGAGTACGGTGTGCCTCTCCAAAGCCCGACCAC |  |
| Hi-TB-R | GAGTTGGATGCTGGATGGCTGCCCCGTCCTCCTCCG |  |
| SLR1-g1-OT1hf | ggagtgagtacggtgtgcCCTCGGCTGCCTCATCTTCCT | Detection of off-target editing |
| SLR1-g1-OT2hf | ggagtgagtacggtgtGCGGTCGCGCGGTCAGC |  |
| SLR1-g1-OT3hf | ggagtgagtacggtgtGCATCGTCGTCGTCTCCC |  |
| SLR1-g2-OT1hf | ggagtgagtacggtgtgcACAGACAAATTCCTGTGACG |  |
| SLR1-g2-OT2hf | ggagtgagtacggtgtgcGACGTGCTCATGGACTGCAAC |  |
| SLR1-g2-OT3hf | ggagtgagtacggtgtgcGAGGTGGACAGCACGGGCTTC |  |
| SLR1-g3-OT1hf | ggagtgagtacggtgtgcAACTAACCGTATACACAAAGC |  |
| SLR1-g3-OT2hf | ggagtgagtacggtgtgcATGCGTGACGACCTAATAGGC |  |
| SLR1-g3-OT3hf | ggagtgagtacggtgtgCAAATGCAAGTGGATATTGGC |  |
| ALS1-g1-OT1hf | ggagtgagtacggtgtgCATTTTCTCCCTTGTTCGCAT |  |
| ALS1-g1-OT2hf | ggagtgagtacggtgtgcGTAAGGTCGATGAGCTTGGCA |  |
| ALS1-g1-OT3hf | ggagtgagtacggtgtGCCAAGAGTACAGTGAATTGGT |  |
| EPSPS-g1-OT1hf | ggagtgagtacggtgtgcAATTAACTAGTAAATGTACCATCGC |  |
| EPSPS-g1-OT2hf | ggagtgagtacggtgtgcAGGTCGTCATCCATCGTTCCT |  |
| EPSPS-g1-OT3hf | ggagtgagtacggtgtgcACGGCAGAGGTGTAGATGGAG |  |
| TB1-g1-OT1hf | ggagtgagtacggtgtgCTACATCAGGCAGTCATCAGC |  |
| TB1-g1-OT2hf | ggagtgagtacggtgtgcTGGCGTCTCGACAGGATCAGC |  |
| TB1-g1-OT3hf | ggagtgagtacggtgtgcTCAAACATCCGATGTGACACC |  |
| SLR1-g1-OT1hr | gagttggatgctggatGGAGGTGGTTTTGTCCAACGT |  |
| SLR1-g1-OT2hr | gagttggatgctggatggTCCTCTCCTCCCGGCCCACT |  |
| SLR1-g1-OT3hr | gagttggatgctggatggATGCGGCACCTTCTGTAATGC |  |
| SLR1-g2-OT1hr | gagttggatgctggatggCGACCCCTGAGGTTGAGATAG |  |
| SLR1-g2-OT2hr | gagttggatgctggatggATTCCGATGTCGTATTCATGC |  |
| SLR1-g2-OT3hr | gagttggatgctggatggCCATCGCCAAAGGTAACTAAATCC |  |
| SLR1-g3-OT1hr | gagttggatgctggatggCCATATCTCAGCTCGTTGACA |  |
| SLR1-g3-OT2hr | gagttggatgctggatggCTATCCGTTGAAGTTGAAGGC |  |
| SLR1-g3-OT3hr | gagttggatgctggatggTACACGACCATACTGCAGGG |  |
| ALS1-g1-OT1hr | gagttggatgctggatggCCAGGCAGTCGCTCGATTCAT |  |
| ALS1-g1-OT2hr | gagttggatgctggatgGAAAATAGAGATAAGACTCAGACAGA |  |
| ALS1-g1-OT3hr | gagttggatgctggatggCAAGTATGATTATTATGGTGATGATGG |  |
| EPSPS-g1-OT1hr | gagttggatgctggatggATGCATCCTCACCTCGTCACC |  |
| EPSPS-g1-OT2hr | gagttggatgctggatgGAGAGATGAGATACATAACGGT |  |
| EPSPS-g1-OT3hr | gagttggatgctggatggTCCGTCGATCTCCGGCGAACA |  |
| TB1-g1-OT1hr | gagttggatgctggatggCGATCCAGGGTGAGAGCT |  |
| TB1-g1-OT2hr | gagttggatgctggatgGCGTTGATGGCCTCGTACAG |  |
| TB1-g1-OT3hr | gagttggatgctggatgGATCACATGATGTCATTATGTTTGG |  |
| HYG-F1 | TTTATCGGCACTTTGCATCGG | Detection of transgene-free plantlets |
| HYG-R1 | CCGTCAGGACATTGTTGGAGC |  |
